# Optimizing scaffold pore size for tissue engineering: insights across various tissue types

**DOI:** 10.3389/fbioe.2024.1444986

**Published:** 2024-11-12

**Authors:** Fariza Mukasheva, Laura Adilova, Aibek Dyussenbinov, Bota Yernaimanova, Madi Abilev, Dana Akilbekova

**Affiliations:** ^1^ Department of Chemical and Materials Engineering, School of Engineering and Digital Sciences, Nazarbayev University, Astana, Kazakhstan; ^2^ Department of Analytical, Colloid Chemistry and Technology of Rare Elements, Al-Farabi Kazakh National University, Almaty, Kazakhstan

**Keywords:** tissue engineering, scaffolds, pore size, porosity, skin tissue, lung tissue, bone tissue, cardiovascular tissue

## Abstract

Scaffold porosity is a critical factor in replicating the complex *in vivo* microenvironment, directly influencing cellular interactions, migration, nutrient transfer, vascularization, and the formation of functional tissues. For optimal tissue formation, scaffold design must account for various parameters, including material composition, morphology, mechanical properties, and cellular compatibility. This review highlights the importance of interconnected porosity and pore size, emphasizing their impact on cellular behavior and tissue formation across several tissue engineering domains, such as skin, bone, cardiovascular, and lung tissues. Specific pore size ranges enhance scaffold functionality for different tissues: small pores (∼1–2 µm) aid epidermal cell attachment in skin regeneration, moderate pores (∼2–12 µm) support dermal migration, and larger pores (∼40–100 µm) facilitate vascular structures. For bone tissue engineering, multi-layered scaffolds with smaller pores (50–100 µm) foster cell attachment, while larger pores (200–400 µm) enhance nutrient diffusion and angiogenesis. Cardiovascular and lung tissues benefit from moderate pore sizes (∼25–60 µm) to balance cell integration and nutrient diffusion. By addressing critical design challenges and optimizing pore size distributions, this review provides insights into scaffold innovations, ultimately advancing tissue regeneration strategies.

## Graphical Abstract

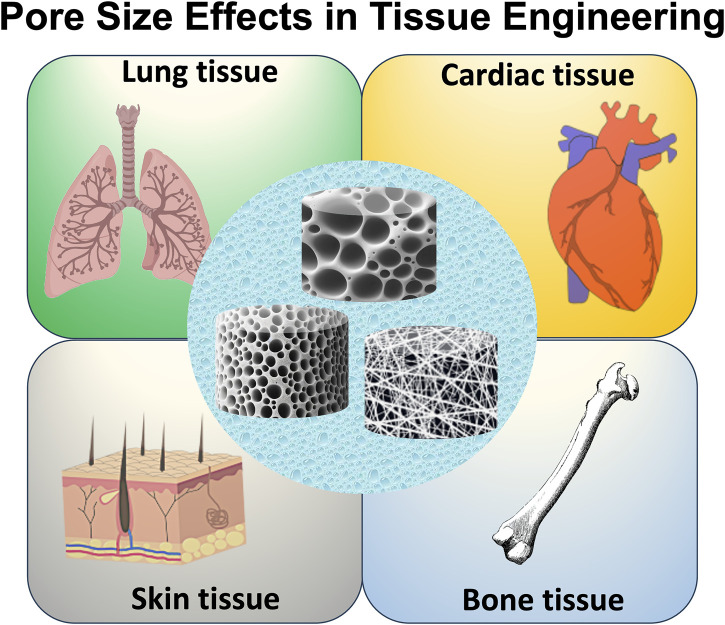

## 1 Introduction

Tissue engineering (TE) applications involve using three-dimensional (3D) scaffolds, which are crucial for creating appropriate microenvironments. This environment is conducive to integrating cells and growth factors, thereby facilitating the regeneration of impaired tissues or organs. The ability of scaffolds to emulate the natural *in vivo* microenvironment is essential because it dictates cellular interactions and responses to mechanical stimuli from their 3D surroundings. Therefore, the material properties of these scaffolds are essential for influencing cellular activity and tissue formation outcomes. The 3D scaffolds are typically characterized by high porosity and interconnected pore morphology. Optimization of scaffold interconnected porosity and pore sizes is essential for facilitating cellular infiltration, migration, vascularization, and efficient diffusion of nutrients, oxygen transport, and waste removal (Hernandez and Woodrow, 2022). Moreover, along with biocompatibility, artificial 3D scaffolds must exhibit sufficient mechanical robustness and temporal integrity to withstand external loading stress and degradation in an aqueous environment.

Several reviews over the past few years have discussed scaffold fabrication methods (Loh and Choong, 2013; Akilbekova et al., 2017, 2023) and the role of inner morphology in biological effects and cellular behavior for TE applications, yielding diverse conclusions for different tissues and organs (Abbasi et al., 2020; Alkentar et al., 2022; Bartoš et al., 2018; Bružauskaitė et al., 2015; Farazin et al., 2023; Flores-Jiménez et al., 2023; Jafari et al., 2017; Kilic Bektas et al., 2018; Liu et al., 2023; Lutzweiler et al., 2020; Murphy and O’Brien, 2010; Perez and Mestres, 2016; Pok et al., 2013; Tytgat et al., 2020; Xia and Luo, 2022; Yadav et al., 2020; Zhang et al., 2022a; Zhang et al., 2022b; Zhong et al.,2020). These studies shed light on the diverse strategies employed to optimize scaffold design and the relationship between scaffold properties, such as morphology, mechanical strength, and cellular behavior, including attachment, proliferation, and differentiation. Although the surveyed papers offer valuable insights into various aspects, such as the selection of scaffold materials and design for different tissue types, it seems that there is no consensus on the optimal pore size to be used for each due to the complexity in the balance between mechanical and biological effects induced by the pore sizes at different phases of tissue formation.

This review provides a comprehensive overview of current advances concerning the influence of scaffold pore size and porosity on guiding tissue formation. We summarized recent studies on skin, bone, cardiovascular, and lung tissue engineering applications, highlighting the complexities associated with developing an ideal scaffold, emphasizing the major parameters for facilitating tissue development, and the role of the pore size in these processes. These specific tissues were chosen due to their varied porosity characteristics, highlighting pore size’s critical role in their respective regenerative processes. The optimal pore size in tissue-engineered scaffolds for a specific tissue type was also discussed in each section.

## 2 The role of pore size in tissue engineering

Porosity, interconnectivity, and pore size play a crucial role in tissue regeneration due to their direct impact on cellular infiltration, proliferation, nutrient and oxygen diffusion, extracellular matrix (ECM) formation, and vascularization (Bartoš et al., 2018; Murphy and O’Brien, 2010). Pores’ spatial distribution and geometry are critical determinants of cellular penetration, attachment, proliferation, and differentiation capabilities and, therefore, directly affect ECM deposition, vascularization, mineralization (in bone TE), and, consequently, functional tissue formation. Scaffolds of variable materials and inner morphology with a wide range of pore sizes (from sub-micron to hundreds of micrometers) were demonstrated to form tissues of different types successfully. To exemplify the influence of pore size on tissue development at various stages, we will examine key findings from bone tissue engineering studies utilizing synthetic scaffolds. In bone regeneration, porous scaffolds have been shown to significantly enhance osteogenesis compared to nonporous solid implants (Kuboki et al., 1998).

Furthermore, a higher cell concentration was observed in the scaffolds with relatively small pores, while cell migration occurred more rapidly within the scaffolds with larger pores (Akay et al., 2004). Also, large pores facilitated vascularization and high oxygenation, showing better efficacy in integration on the bone-implant interface due to unimpeded nutrient and oxygen transport (Abbasi et al., 2020; Lutzweiler et al., 2020; Murphy and O’Brien, 2010). In contrast, smaller pores were shown to promote osteochondral ossification (Karageorgiou and Kaplan, 2005). On the other hand, exceptionally large pores that exceed a few hundred microns were demonstrated to enhance angiogenesis and higher efficiency of bone ingrowth along with enhanced chondrogenesis and alkaline phosphatase levels, affecting cell shape, which may indirectly indicate stress and general condition of cells in a given environment. Scaffolds with a 50–700 µm pore size range are commonly used in bone tissue engineering. This range represents attempts to balance between biological aspects and physical properties of the construct during tissue growth, as pore sizes were shown to impact the scaffold’s structural integrity and degradation rate (Alkentar et al., 2022; Bružauskaitė et al., 2015; Perez and Mestres, 2016; Zhang et al., 2022a).

In comparison, Yannas et al. demonstrated that skin regeneration on a porous scaffold was feasible only with pore sizes ranging from 20 to 120 µm (Yannas et al., 1989). Like bone tissue, O’Brien et al. found that cell adhesion depends on the available surface area, demonstrating a decrease in cell adhesion as pore diameter increases (O'Brien et al., 2005). While scaffolds with pores of 20–50 µm demonstrate improved attachment of cells to the scaffold, larger pore diameters enable better fibroblast migration, enhancing vascularization and facilitating nutrient transfer while maintaining dermis formation integrity in a skin substitute (Kilic Bektas et al., 2018). Successfully engineered cardiac tissue is characterized by well-organized vascular networks that supply oxygen and nutrients to the cells. This fact imposes specific requirements on the scaffold’s pore sizes to ensure both capillary formation and the infiltration of endothelial cells. Therefore, scaffolds of high porosity and pore sizes ranging from 20 to 300 µm are frequently used to facilitate unimpeded vascularization as a priority (Pok et al., 2013; Rouwkema et al., 2008). Similarly, high porosity scaffolds with a lower pore size range are commonly used for lung tissue engineering to promote vascular structure formation and blood circulation in the lung tissue.

Optimizing pore size or narrowing the range of pore sizes can significantly impact tissue engineering by customizing the scaffold’s structure to meet the specific needs of different tissues (Abbasi et al., 2020; Perez and Mestres, 2016; Zhang et al., 2022a). Selecting a scaffold with the optimal pore size for a particular tissue type can balance cellular attachment, migration, and nutrient transport. This improves the predictability of scaffold performance and enhances tissue formation and regeneration. Such optimization efforts can lead to more effective scaffold designs, facilitating faster healing, better integration with host tissue, and improved functionality in tissue-engineered constructs.

While this review primarily focuses on optimizing scaffold pore size, it is important to acknowledge that other critical properties, such as pore shape and geometry (Flores-Jiménez et al., 2023; Zhao et al., 2021), interconnectivity (Calore et al., 2023; Jia et al., 2021), mechanical properties (Chao et al., 2021; Sabree et al., 2015), biodegradability (Martin et al., 2014; Wang et al., 2017), microarchitecture (Cavo and Scaglione, 2016; Eichholz et al., 2022), and surface chemistry (Dave and Gomes, 2019; Zhao et al., 2016) can notably influence scaffold performance and tissue formation.

## 3 Pore size in skin tissue engineering

### 3.1 Porous anatomical features of the skin

The skin, an essential barrier and regulatory organ, plays critical roles in preventing infection, mitigating mechanical damage, and controlling moisture and heat loss, thereby regulating the body temperature. It comprises three distinct layers: epidermis, dermis, and hypodermis.

The epidermis, the outermost layer, consists of five tightly interconnected stratified sublayers of keratinocytes and lacks blood or lymphatic vessels. It relies on the diffusion from the underlying dermis for nutrient acquisition, oxygenation, and waste removal. The thickness of the epidermis varies significantly, ranging from 30 to 600 µm. Beneath the epidermis lies the dermis, characterized by two layers with unique structures. The upper layer, known as the stratum papillare, exhibits a spongy composition, primarily consisting of loosely woven collagen fibers and ECM, which facilitates extensive vascularization. This layer also projects to the epidermis to enhance the exchange of nutrients and oxygen. The deeper layer, the stratum reticulare, is distinguished by its dense network of elastic and collagen fibers, imparting the properties of firmness, extensibility, and elasticity to the dermis. The dermis varies in thickness from 2 to 6 mm. The innermost layer, the hypodermis or subcutaneous layer, anchors the dermis to underlying musculoskeletal structures. This well-vascularized, loosely textured layer is rich in large nerves, blood vessels, connective tissue, and predominantly white adipose tissue.

**FIGURE 1:**
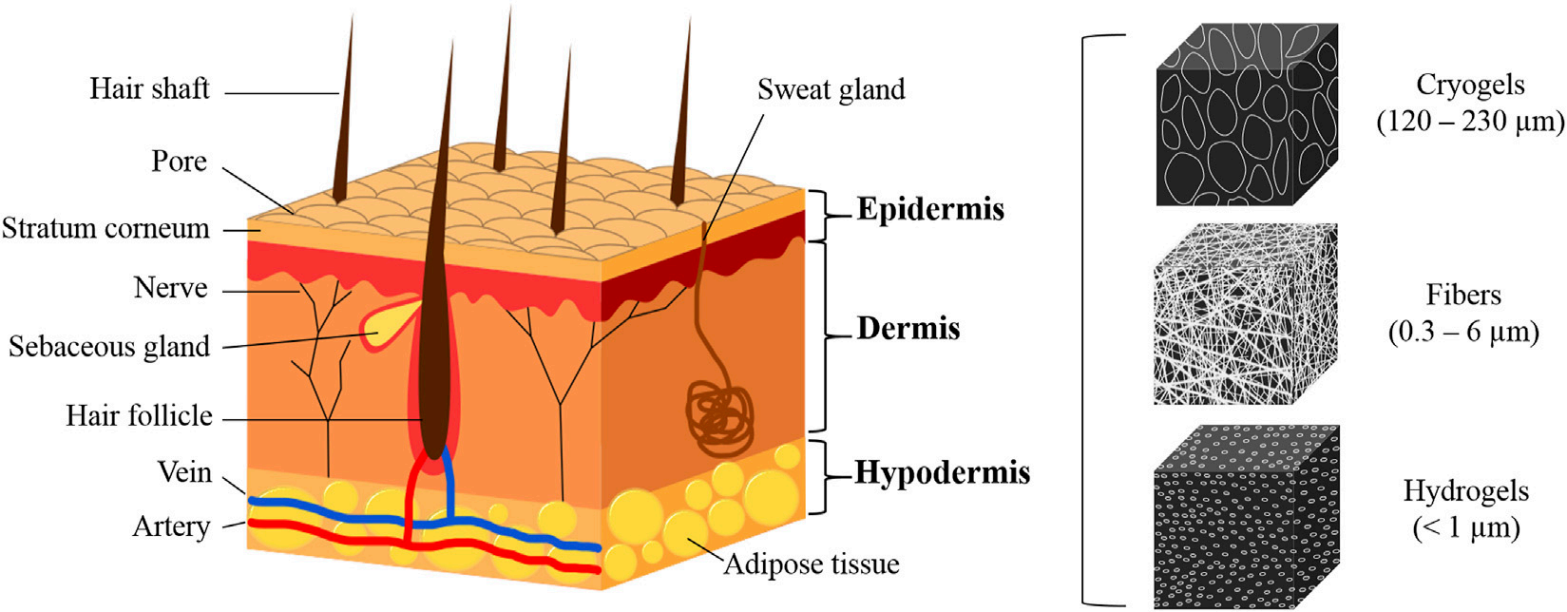
Schematic of the skin tissue structure and mimicking materials.

To replicate skin grafts for therapeutic applications, it is essential to emulate these distinct layers (Figure 1). The epidermis-analogous layer should primarily host keratinocytes, whereas the dermal substitute should incorporate a spongy structure conducive to fibroblast habitation and extensive vascularization, with increased density at the deeper part. Furthermore, the bottom layer, resembling the hypodermis, should have a loosely textured framework supporting larger nerves and blood vessels. Critical to the design of such bioengineered scaffolds is the incorporation of a porous architecture aptly tailored for the seeding and attachment of diverse cells, including keratinocytes, fibroblasts, and hair follicle bulge stem cells (HFBSCs), with pore sizes ideally ranging between 5 and 18 µm. Additionally, scaffolds should guide cellular responses, including collagen formation and organization, mimicking the behavior seen in functional tissues (Akilbekova et al., 2018; Boddupalli et al., 2020). Achieving a normal human dermal tissue porosity distribution and average pore diameter can help fabricate artificial dermal scaffold materials.

### 3.2 Recent advances in mimicking skin tissue

Scaffolds with small pore sizes are commonly used to replicate skin tissue structures and serve as models for wound healing. Kumar et al. (2022) developed a three-layered electrospun scaffold using a dual protein-based blend of silk fibroin and silk sericin, combined with PVA and silver sulfadiazine as an antibacterial agent. The multilayered structure was designed to mimic the epidermis, dermis, and subcutaneous tissues, giving the scaffold distinct morphologies that closely resemble native skin architecture. This design enables the scaffold to replicate the transport properties of natural skin. The upper and middle layers featured thin fibers, approximately 170–230 nm in diameter, with interfiber pore sizes around 1 µm as shown by SEM images. The bottom layer comprised thicker fibers (about 1.5 µm in diameter) with internal pores of around 0.3 µm for drug release and interfiber pores ranging from 2 to 4 µm. The materials and sub-micrometer pore sizes of the nanofibrous scaffold led to excellent wettability and a controlled degradation rate, with 48% weight loss observed after 18 days *in vitro*. *In vivo* testing on mouse models showed 95% wound closure by day 12, significantly outperforming the control group, which achieved only 68% closure. Micro-CT imaging and histological analysis confirmed accelerated wound healing and the formation of new skin tissue, with the scaffolds promoting the development of the epidermis, dermis, blood vessels, and hair follicles. Despite the small pore size, the study demonstrated adequate cell infiltration, proliferation, ECM secretion, and the regeneration of skin structures, including the epidermis, dermis, and hair follicles. However, the authors primarily focused on the impact of drug concentration and its release on cell viability and wound healing, without addressing how such a dense electrospun mesh facilitated fibroblast infiltration and proliferation, given that fibroblast dimensions are significantly larger than the fabricated pores. This aspect remains unexplained and warrants further investigation.

In contrast, using a freeze-drying synthesis approach, Yang et al. (2019a) fabricated a highly porous scaffold from a silk fibroin/hyaluronic acid/sodium alginate polymer mixture. The obtained scaffold, with an average pore diameter of 93 μm, demonstrated negligible degradation of ca. 2% weight over 15 days. Yet the fabricated scaffolds, seeded with NIH-3T3 fibroblasts, showed decent cell attachment, proliferation, and growth. *In vivo* studies on mice showed improved re-epithelization, significant ECM remodeling, and enhanced angiogenesis.

It should be noted that some works report fabrication of scaffolds with extremely large pore sizes, 200–400 μm, and their successful implementation for skin tissue regeneration applications, including cell attachment, proliferation, ECM deposition, and angiogenesis (Chu et al., 2021; Ramasamy et al., 2021). However, due to the ambiguous definition of pores (Trifonov et al., 2024), one should pay attention that the reported pore size refers to the distance between the fibers (voids) created by 3D printing deposition, whereas interconnected, inner-morphology interconnected porosity of nanofiber-based polymer material, used for the fabrication of scaffolds is not reported.

One of the popular and successful strategies for mimicking natural skin layers is the fabrication of multilayered scaffolds with different pore sizes that fit the structural properties of separate skin layers. Zandi et al. fabricated a bilayered, Laponite-doped scaffold using GelMA hydrogel and electrospun gelatin nanofibers for wound treatment and skin regeneration applications, with average pore sizes of ca. 12 and 2 μm, respectively, to mimic different skin layers and thus to increase the efficacy of mass transport and maintaining barriers for undesired infiltration of cells (Zandi et al., 2021). The pore size gradient also enables the seeding of cells of different types and sizes on the scaffold, and the interconnection of the pores allows for better cell-cell interactions and formation of the distinct epidermal, dermal, and reticular layers. Sumathy and Nair (Sumathy and Nair, 2020) developed a trilayer gelatin-PEG dimethacrylate scaffold with graded interfiber pore sizes corresponding to different skin layers. In this case, variability in polymer concentration controlled the resulting pore sizes. The obtained pores showed relatively minor differences in diameter, ranging from ca. 1.5–2.2 µm, and were populated with keratinocytes, hair follicle bulge-derived stem cells (HFBSCs), and fibroblasts. Histology showed that the fabricated structure supports the formation of continuous epidermal and distinct dermal layers.

Larger pore sizes are generally beneficial for promoting vascularization and enhancing nutrient transport within scaffolds. This concept is based on diffusion principles and the relative sizes of nutrients, cells, and bioactive molecules, such as vascular endothelial growth factor (VEGF) and fibroblast growth factor (FGF), which must efficiently diffuse through the scaffold to reach cells deep within the structure. Fibroblasts can migrate without compromising dermal formation in skin substitutes with pore diameters >100 µm (Kilic Bektas et al., 2018). However, cell migration in the scaffolds with pore diameters >200 µm is too extensive and leads to a lower cell density. Incorporation of nanofibrous membranes with sub-micron pores in the bottom layer was shown to have a positive effect on cell adhesion, significantly increasing collagen formation and angiogenesis, outperforming the scaffold without the membrane (Tavakoli et al., 2023).

Furthermore, additional architectural parts can be added to mimic the dermal layer more accurately. Micro-pits with 40–100 µm in diameter were embedded into the scaffolds to form hair follicles (Sumathy and Velayudhan, 2023). A bilayer nanofibrous scaffold was fabricated, consisting of a top layer with a pore size of 2.5 µm and a bottom layer with a pore size of 1.5 µm, featuring complementary micro-pits. The top layer was seeded with keratinocytes, the bottom layer was seeded with dermal fibroblasts, and the HFBSCs were embedded in the micropits. The results indicated the formation of distinct epidermal and dermal layers with HFBSCs in the micro-pits by day 14. Choi et al. (2021) fabricated electrospun polycaprolactone (PCL)/keratin multilayered scaffold on 3D printed support for skin tissue regeneration applications. The resulting scaffold consisted of microfibrous, with pore diameters ranging from 4.5–6.4 µm, and nanofibrous membranes with sub-micrometer pores. Human dermal fibroblasts were seeded on the microfibrous parts, and after 3 days, human immortalized keratinocytes were added to the nanofibrous layers. The scaffold demonstrated excellent cell attachment and prevention of cell infiltration between the domains, generating distinct epidermal and dermal layers. Also, the authors reported very high stability of the scaffold, demonstrating only ca. 10% degradation by weight after 30 days.

Table 1 summarizes the studies focusing on the development of advanced skin tissue engineering scaffolds, emphasizing the role of pore size in facilitating skin regeneration.

**TABLE 1:** Summary of representative studies on skin tissue generation within porous scaffolds.

Cell type	Preparing method	Materials	Pore size	Results	Ref.
NIH-3T3 fibroblasts	Electrospinning	Silk fibroin, Silk sericin, PVA, PCL	0.3 μm	The microporous layer prevents bacterial invasion, and the nanofibers enhance fibroblast adhesion and proliferation, wound closure, and skin tissue formation	Kumar et al. (2022)
NIH-3T3 fibroblasts	Freeze-drying	Silk fibroin, Hyaluronic acid, Sodium alginate	∼93 μm	The scaffold facilitated cell adhesion and proliferation, supported increased collagen deposition, and demonstrated the fastest wound healing rate, with enhanced angiogenesis and the formation of new epidermis closely aligned with the neonatal dermis layer	Yang et al. (2019a)
HUVEC, Human Splenic Fibroblasts, HaCaT	SLg peptide, GelMA	3D printing, freeze-drying	200–400 μm	Inflammatory cells filled the pores, where later collagen fibers and blood vessels were formed, and thus, led to the growth of skin tissue	Chu et al. (2021)
Fibroblasts, Keratinocytes	PCl, collagen	3D bioprinting	195.8 ± 9.1 µm	The scaffold supported growth of fibroblasts and keratinocytes and further formation of dermal and epidermal layers with tight junction between them	Ramasamy et al. (2021)
No cell	Electrospinning, freeze-drying	Gelatin, GelMA/Laponite	∼10 μm	The bilayer scaffold, made of hydrogel and nanofibers, showed superior performance in wound closure, tissue granulation, and skin appendage formation compared to the individual layers	Zandi et al. (2021)
HFBSCs, keratinocytes, fibroblasts	Electrospinning	Gelatin, PEGDMA	1.6–2.5 μm	Small pore sizes regulate keratinocyte growth and promote epidermis formation	Sumathy and Nair (2020)
Human keratinocytes and fibroblasts	Freeze-drying	NaCMC, Collagen	75–123 μm	Keratinocytes in small pores and fibroblasts in large pores achieved paracrine signaling, but the high-water content in the large pores deteriorated fibroblast attachment	Kilic Bektas et al. (2018)
L929 fibroblasts	Electrospinning, freeze-drying	PAAm, Aloe vera (powder)	∼230 μm	The combination of macropores and nanopores supported fibroblast proliferation, collagen secretion, angiogenesis, and the formation of epidermal and keratin layers	Tavakoli et al. (2023)
HFBSCs, keratinocytes, fibroblasts	Electrospinning, casting	Gelatin-PEG Methacrylate, PVA	1.5–2.5 μm	Gradient in pores size facilitates the targeted delivery of distinct cell types specific to each skin layer. The micro-pits aid in formation of skin appendages	Sumathy and Velayudhan (2023)
Human dermal fibroblasts, human epidermal keratinocytes	Electrospinning, 3D printing	PCL, keratin	4.5–6.4 μm	Fibroblasts demonstrated enhanced growth on microfibers, whereas keratinocytes proliferated better on nanofibers with a smaller pore size	Choi et al. (2021)

### 3.3 Critical evaluation: the optimal pore size for STE

Based on the literature discussed, we suggest that the ideal scaffold structure used for skin tissue regeneration involves a multilayered design with variable pore sizes that replicate the distinct characteristics of the epidermal and dermal layers of the skin. The specific demands for each layer and the biological processes involved in skin regeneration support the need for carefully tailored pore sizes. Pore gradients within scaffolds enable better organization of various cell types, promoting the creation of distinct epidermal and dermal layers while maintaining the functional integrity of the growing tissue. Small pore sizes, typically in the sub-micrometer to 2 µm range, are beneficial for mimicking the epidermis, promoting cell attachment, and facilitating organized skin layer formation. Moderate pore sizes, around 2–12 μm, are more suited to the dermal layer, allowing for proper cell migration, nutrient transport, enhanced deposition of ECM, and organization of skin structures. Larger pores, typically in the range of 40–100 μm, are favorable for supporting specialized structures such as blood vessels and hair follicles. Based on the reviewed works, we do not see any sizable advantages in using scaffold layers with pores that significantly exceed the diameter of 100 µm. A balanced, multilayered scaffold design with tailored pore sizes for different skin layers offers the most promising approach to achieving complete and functional skin regeneration.

However, it is important to note that the existing literature has not systematically assessed the effects of different pore sizes across varying conditions and cell types. To derive more balanced conclusions, future research should focus on conducting controlled studies that systematically evaluate the impact of pore size variations on skin regeneration outcomes. Investigating how different pore sizes interact with various cell types, growth factors, and physiological conditions could provide valuable insights into optimizing scaffold design for enhanced functionality and efficacy in skin tissue engineering.

## 4 Pore size in bone tissue engineering

### 4.1 Porous anatomical features of bone tissue

**FIGURE 2:**
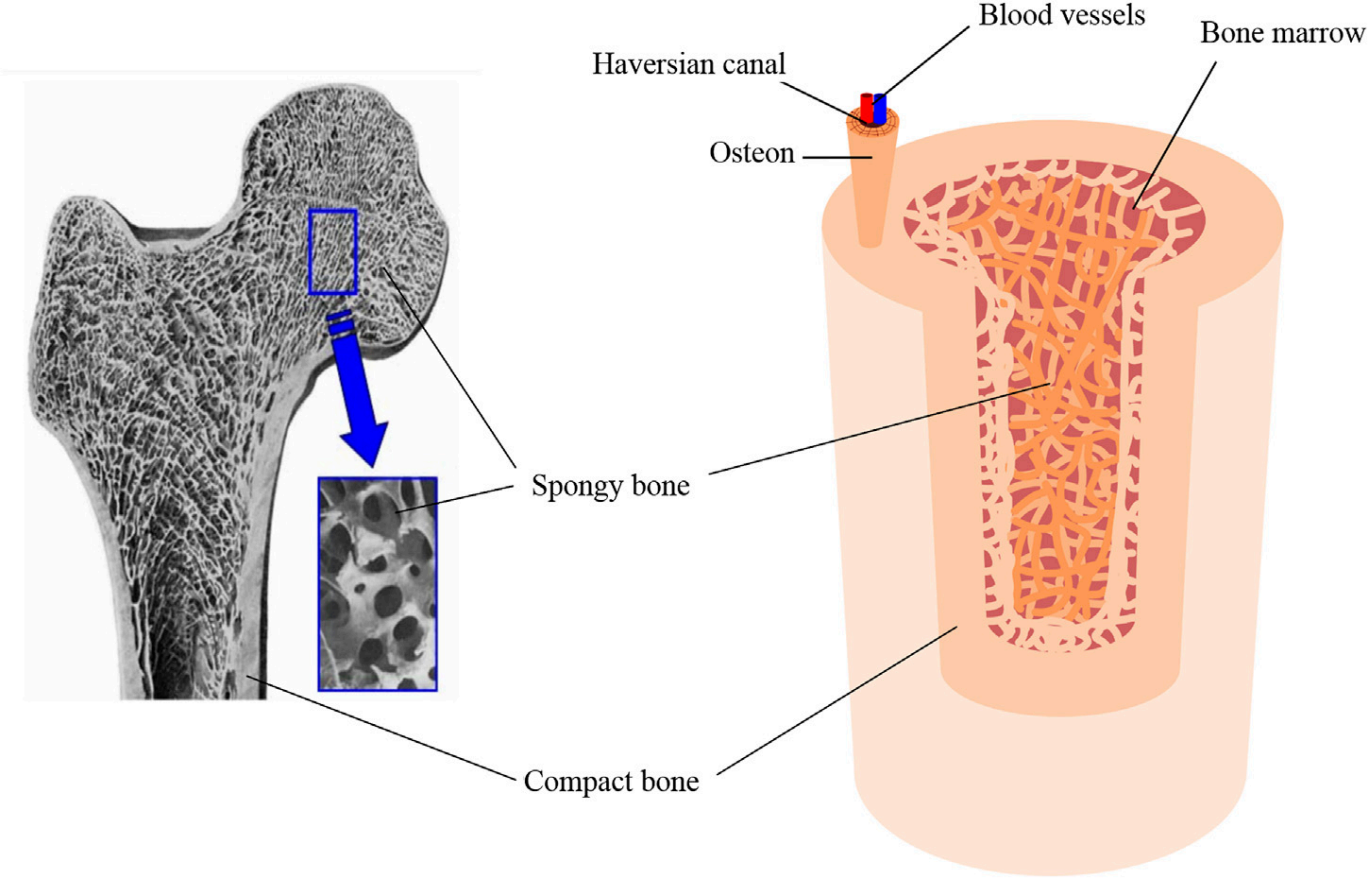
Scanning electron micrograph (SEM) of bone, reproduced with permission from Ishikawa (Ishikawa, 2010), copyright 2010, MDPI, and schematic of the bone tissue structure.

Bone tissue engineering endeavors to replicate the intricate structures of compact and cancellous bones to enhance bone regeneration and integration. Compact (cortical) bone forms the outer layer of bone tissue and is characterized by its dense, tightly packed structure. This layer is primarily composed of osteons and the Haversian system, which includes Haversian canals. These canals are essential for housing blood vessels and nerves, facilitating the supply of nutrients, and removing waste products. Compact bone plays a vital role in the skeletal system’s overall durability and stability due to its mechanical strength and resistance to compression. The inner layer, known as cancellous (spongy) bone, plays a vital role in the metabolic activity of the tissue. This layer comprises a network of trabeculae, which are interconnected struts of bone that provide structural support while also allowing the adaptability of bones to mechanical changes. The porous nature of cancellous bone enables it to house a significant number of blood vessels, ensuring an efficient nutrient supply to bone tissue (Figure 2). Within this spongy matrix, bone marrow is a crucial element for blood formation (Cooper et al., 2013).

The porosity and pore size of bone tissues significantly influence their structural integrity and functionality. Compact bone porosity primarily arises from the Haversian system, with canals typically ranging from to 20–100 µm in diameter, interconnected by Volkmann’s canals (Jones et al., 2004). The spaces between the lamellae, known as lacunae and house-osteocytes, are connected by canals called canaliculi. Canaliculi, which are at the submicron level, allow for the exchange of nutrients and waste products between the blood vessels and osteocytes. These microscopic channels contribute to overall porosity and are critical for bone metabolic activity (Smit et al., 2002). Cancellous bone pores, formed by spaces between trabeculae, vary widely in size depending on their location within the body and the specific function of the bone. The average pore diameter within the bone depends on multiple factors, e.g., bone function, age, and patient condition. It can vary from one individual to another, yet in general, the range spans between 10–50 μm and 300–600 μm, for cortical and trabecular regions, respectively (Currey, 2002).

### 4.2 Recent advances in mimicking bone tissue

Recent activities in bone tissue engineering (BTE) have concentrated on increasing the effectiveness by creating scaffolds that mimic the natural bone structures, addressing the critical requirements of biocompatibility, unimpeded mass transfer, appropriate degradation rates, mechanical stability, and promotion of bone growth and blood vessel formation (Söhling et al., 2020; Xue et al., 2022). Some studies and review articles suggest that efficient mass transfer, cell migration, and vascularization require scaffolds with open, porous structures and pore diameters in the millimeter range (Malda et al., 2007; Henkel et al., 2013) to support diffusion across the scaffold and prevent hypoxic conditions. Other sources, however, identify an optimal pore size range of 100–600 µm (or in some cases, 200–600 µm) (Abbasi et al., 2020; Palaveniene et al., 2019; Zou et al., 2016). Other works highlight that such large pore dimensions can majorly impair scaffold stability and significantly reduce cell attachment. Therefore, interconnected pores with dimensions of tens to hundreds of micrometers should also be considered to enhance cell attachment and increase seeding efficiency (Einhorn and Gerstenfeld, 2015; Loi et al., 2016).

For example, Cidonio et al. (2019) fabricated a Laponite/gellan gum scaffold with interconnected homogeneous porosity with a diameter of ca. 100 μm. They reported excellent attachment and cell density of mouse promyoblast C2C12 (able to differentiate into osteoblasts) seeded on the scaffold, with angiogenetic evidence, yet a small number of penetrating vessels *in vivo*. Gayer et al. (2019) fabricated Polylactide/calcium carbonate scaffolds via selective laser sintering with interconnected pores in the range of 20–120 µm to facilitate cell attachment, and the designed open channels of ca. 1 mm in diameter to increase its potential for angiogenesis and unimpeded transport of nutrients. The authors reported good biocompatibility of the material using MG-63 osteoblast-like cells and highlighted excellent mechanical properties of the scaffold, comparable with those of the native human bone (up to 100 MPa), without providing any data on scaffold degradation rate, and its bio-inductive properties, including seeding efficiency, cell distribution, proliferation, ECM deposition or vascularization. Demir et al. (2018) fabricated Sr-doped chitosan/montmorillonite scaffold with 45–75 μm pores, demonstrating its high efficacy for osteoblasts attachment, proliferation, and bioactivity over 14 days, yet with no evidence for efficient ECM deposition or vascularization. Shahin-Shamsabadi et al. (2018) developed a PCL/Zn-modified bioactive glass scaffold with excellent mechanical properties, reaching Young’s modulus of ca. 45 MPa and pores <100 μm, evidenced by SEM images. The authors reported decent attachment of MG-63 cells to the scaffold and their enhanced mineralization, yet no evidence for vascularization was demonstrated.

In other studies, scaffolds with higher pore diameters but no interconnected porosity were fabricated for bone tissue regeneration applications. For example, a scaffold made of polyurethane foam, with an average pore diameter of ca. 400 μm was modified with hydroxyapatite (HAp) nanoplates, which sizably increased the roughness of the material, creating a porous surface with pore diameter <10 μm (Meskinfam et al., 2018). The fabricated scaffold showed excellent cytocompatibility (ca. 100%), and efficient cell attachment and further bioactivity, yet no evidence of ECM deposition or vascularization *in vivo* was demonstrated. Liu et al. (2019) used PCL/Sr-modified HAp 3D printed scaffold with open channels of ca. 200 μm diameter for bone regeneration. They showed evidence of efficient cell attachment and their further differentiation. However, no evidence of ECM deposition or angiogenesis was demonstrated. On the other hand, a scaffold produced from gelatin/β-tricalcium phosphate (TCP) (Gu et al., 2019), with 400 μm designed voids, along with interconnected pores with a diameter of ca. 10 μm, exhibited excellent mechanical properties, suitable for temporary substitution of native load-carrying bone, and demonstrated exceptional cell attachment, proliferation, differentiation, ECM deposition and evidence of angiogenesis.

Emphasis on close replicating the intricate pore structures of compact and cancellous bones can also be noted. Due to the layered structure of bone tissue, the fabrication of scaffolds comprised of two or more layers with different pore sizes allows evaluation of their role at various phases of osteogenesis. Almela et al. (2017) utilized 3D printing to create β-Tricalcium phosphate-based inks to fabricate bi-layer scaffolds via 3D printing to resemble the natural bone tissue structure, mimicking cortical and cancellous bone regions. The two distinct layers had pore sizes of ca. 240 μm and 410 μm, as a compact and cancellous layer, respectively. Mechanical characteristics of the resulting scaffolds were within the range of human cancellous bone, demonstrating Young’s modulus in an MPa range. *In vitro* results reported good cytocompatibility and enhanced gene expression, indicating the potential of the scaffold to serve as a synthetic graft material. Di Luca et al. introduced hierarchical scaffolds with a tailored radial porosity gradient to approximate the radial structure of bone (Di Luca et al., 2016). The scaffold was fabricated from polyethylene oxide terephthalate/polybutylene terephthalate block co-polymer by extrusion and featured pores of varying sizes (470 µm in the outer ring, 750 µm in the middle zone, and 940 µm in the inner part), resulting in a porosity gradient that aims to mirror the natural bone structure. The authors found that the cell seeding efficiency in the outer region, with the smallest pores, was ca. 7% higher compared to the inner region and reached only ca. 25%. It was also found that pore size and location have a sizable effect on differentiation and calcification, reporting higher Runx-2 and BSP gene expression in the outer region with smaller pores. Another example of a gradient scaffold, characterized by three distinct yet seamlessly interconnected porous regions with pore diameters of ca. 20 μm, 120 μm, and 200 μm, was fabricated from gelatin/oxidized alginate hydrogel compositions (Mukasheva et al., 2024). The resulting scaffold showed superior stability, yet mechanical properties, incomparable with the natural bone, do not allow for the implementation of load-bearing bone regeneration. However, the realization of a pore size gradient allowed demonstrating an excellent attachment and proliferation of rMSC mainly in the small and medium size pores regions, leaving the potential of enhanced vascularization (was not demonstrated) to larger pores. Additionally, it was shown that introducing 20 µm-size pores improves cell seeding efficiency, serving as a trap for the cells and preventing their washing out at the initial stages and improving cell seeding efficiency from ca. 25%–45%.

It should be noted that none of the cited works investigated or optimized the pore sizes systematically.

Table 2 summarizes the studies focusing on the development of scaffolds for bone tissue engineering applications, emphasizing the role of pore size in facilitating bone tissue formation.

**TABLE 2:** Summary of representative studies on bone tissue generation within porous scaffolds.

Cell type	Preparing method	Materials	Pore size	Results	Ref.
Immortalized mouse premyoblast C2C12	3 days printing	Laponite nanoclay/gellan gum in agarose fluid gel	100 µm	The fabricated scaffold, surrounded by the agarose gel demonstrated decent cell viability (80%) and significant increase in cell density over time and enhanced angiogenesis	Cidonio et al. (2019)
Human osteosarcoma cells MG-63	Selective laser sintering	PLA/CC composite powders	20–120 μm interconnected porosity and ca. 1 mm diameter designed voids	The cell culture with MG-63 osteoblast-like cells exhibited biological activity for up to 10 days in all specimens. No information on scaffold degradation	Gayer et al. (2019)
Human primary osteoblasts	Freeze-drying	Strontium-doped chitosan/montmorillonite	45–75 μm	The developed chitosan/montmorillonite scaffolds supported osteoblast attachment, proliferation, and bioactivity over 14 days. No mechanical characterization, cytocompatibility, degradation rate, ECM deposition, or vascularization were demonstrated	Demir et al. (2018)
Human osteosarcoma cells MG-63	Solvent casting/molding	PCL/bioactive Zn-modified glass 58S5Z	<100 μm	The scaffolds exhibited excellent mechanical properties, comparable to load-bearing bones and demonstrated high cell attachment, density, proliferation, and mineral deposition. No degradation rate was reported	Shahin-Shamsabadi et al. (2018)
Bone marrow mesenchymal stem cells	Gas foaming	Polyurethane/hydroxyapatite plates	400 μm, with sub-µm cavities	The fabricated PU scaffolds, deposited with hydroxyapatite demonstrated excellent mechanical properties, cell attachment, viability of ca. 100%, and evidence of biological activity over 14 days. No degradation rate was reported, as well as evidence of ECM deposition or vascularization	Meskinfam et al. (2018)
Bone marrow-derived mesenchymal stem cells (BMSCs)	3 days printing	Polycaprolactone/Strontium-containing hydroxyapatite	Fabricated 200 µm channels with no interconnected porosity	SEM analysis showed well-defined orthogonal structures with MPa range stress values over 40% range of strain. The scaffold allowed unimpeded cell infiltration, attachment on the deposited filaments, cell growth, and osteogenic differentiation over 21 days. No information on the biodegradation rate of the scaffold was provided. No evidence for ECM deposition or vascularization	Liu et al. (2019)
hBMSCs, human umbilical vein endothelial cells	Cryogenic 3D printing	Gelatin/Mg-doped β-tricalcium phosphate	400 μm voids with ca. 10 μm interconnected porosity	The scaffolds are composed of high-roughness filaments and supported cell attachment, migration, demonstrated excellent proliferation evidence of ECM deposition, osteo- and angiogenesis	Gu et al. (2019)
Human osteoblasts	3D bio plotting	β-tricalcium phosphate	242.2 ± 24.3, 410.5 ± 27.9 µm	The bi-layer scaffold replicated the architecture and mechanical properties of the cancellous and cortical parts of bone tissue	Almela et al. (2017)
hMSCs	3D printing	Poly(ethylene oxide therephtalate), Poly(butylene therephtalate)	475 ± 4, 750 ± 26, 939 ± 20 μm	A porosity gradient of the scaffold supported the osteogenic differentiation, gene expression, and secretion of calcium and phosphate mineral deposits	Di Luca et al. (2016)
rat MSCs	3D printing	Gelatin, Oxidized alginate	∼20, 120, 200 μm	The hydrogel scaffold with interconnected gradient pore sizes showed improved mechanical stability, cell-seeding efficiency, osteodifferentiation, and mineralization	Mukasheva et al. (2024)

### 4.3 Critical evaluation: the optimal pore size for BTE

Based on the literature, there is no consensus on the ideal pore sizes and scaffold inner morphology, and summarizing the results, one can find that a broad range of pore sizes (20–1,500 µm) is reported as potentially beneficial for BTE applications. The variability in the recommended pore sizes can be attributed to differences in the research methodologies, materials utilized, and specific biological processes under scrutiny. However, some general conclusions can be derived based on the experimental observations. For instance, the lowest pore size, mentioned in multiple studies with experimental evidence, suggests consideration of pores >100 μm to allow efficient nutrient and waste transport and maintain decent cell migration within the scaffold. Scaffolds with pore sizes below these values provide only limited experimental data and frequently highlight the potential of the fabricated constructs in BTE, without experimentally observing a complete cycle of tissue formation at its different phases (Farazin et al., 2023; Lutzweiler et al., 2020). A relatively broad range of pore diameters is mentioned regarding cell attachment and seeding efficiency, and diameters from 20 to 400 μm are frequently reported. Since the affinity of cells to the scaffold is highly dependent on the material properties rather than on pore size, pores with diameters <100 μm can be efficient for increasing seeding efficiency and enhanced cell attachment (Murphy et al., 2010), yet it is highly recommended combining it with pores of higher dimensions to support tissue development at later stages efficiently, e.g., vascularization. For example, gradient or hierarchical design seems to be a highly efficient approach in scaffold fabrication. It allows for maximizing the osteoconductive and osteoinductive properties of the scaffold. Whereas the upper limit in pore dimensions seems to be very broad and large pores do not impose any biological restrictions (Büyük et al., 2022; Gupte et al., 2018), it is worth considering mechanical aspects of the scaffold when pore sizes of interconnected internal porosity exceed several hundreds of micrometers, which may lead to structural collapse of the scaffold. At the cellular level, osteoblasts, which typically measure approximately 50 μm, prefer larger pore sizes, ranging from 100 to 200 μm, during the regeneration of mineralized bone post-implantation. These dimensions facilitate macrophage infiltration and promote the entry of additional cellular growth factors essential for tissue colonization, migration, and vascularization *in vivo* (Shahin-Shamsabadi et al., 2018; Grémare et al., 2017).

In conclusion, considering the anatomical structure of bone, which includes both dense and spongy regions characterized by varying porosity and pore sizes, as well as experimental findings that demonstrate successful bone tissue formation on scaffolds mimicking natural bone structure, it is evident that multi-layered scaffolds with different pore diameters can offer significant benefits. Smaller pores, 50 < d < 100 μm, promote better cell attachment, seeding efficiency, and proliferation, while larger pores, 200 < d < 400 μm, create optimal nutrient diffusion and angiogenesis conditions. Larger pores facilitate the infiltration of capillaries and arterioles/venules up to 10 and 200 μm, respectively, essential for long-term nutrient delivery, while reducing diffusion resistance and promoting efficient transport to deeper regions of the scaffold. These variations in pore size, influenced by key biological processes such as alkaline phosphatase activity, extracellular matrix deposition, calcification, and angiogenesis, ensure the scaffolds support bone tissue formation effectively. Therefore, a scaffold design that incorporates regions with both smaller and larger pores can provide an ideal environment for bone regeneration. Using scaffolds with pores that exceed the recommended size of ca. 400 μm does not offer significant proven benefits, especially in the case of interconnected porosity, where such large pores can lead to structural and mechanical weakness.

## 5 Pore size in cardiac tissue engineering

### 5.1 Porous anatomical features of cardiac tissue

**FIGURE 3:**
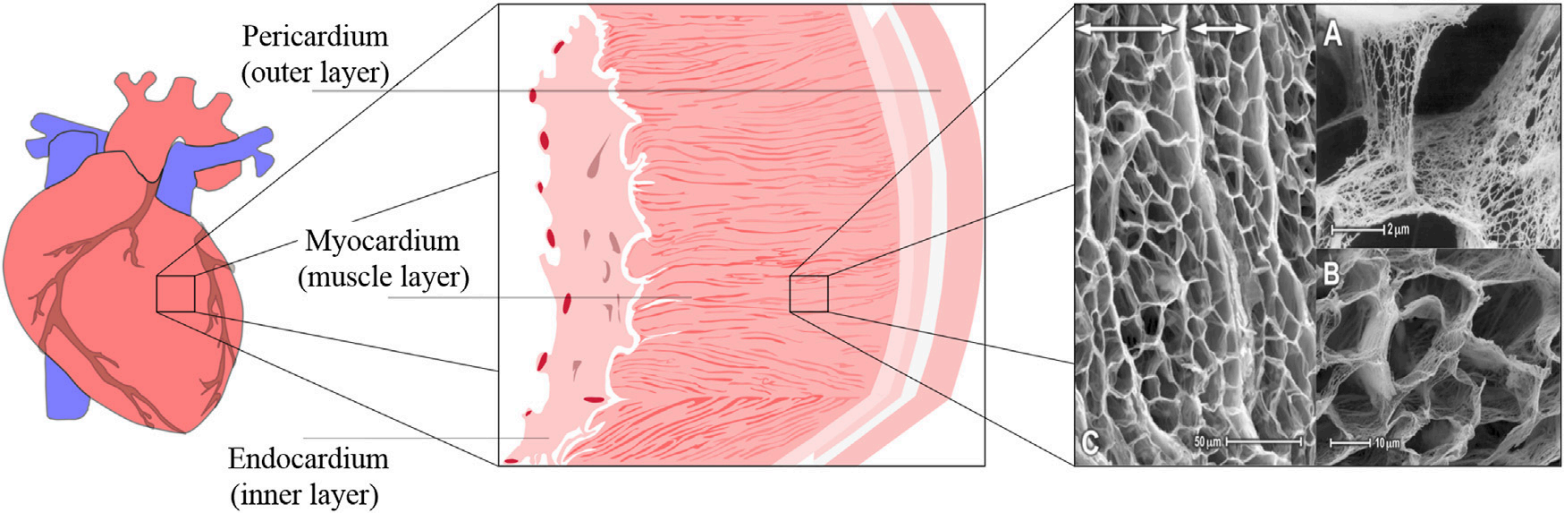
Schematic of the cardiac tissue structure and SEM image of the ECM structure in rabbit and canine myocardium. The SEM image was reproduced with permission from Nordsletten et al. (2021), Copyright 2021, Elsevier.

Heart failure, a major cause of global mortality, stems from the heart’s inability to pump blood effectively, often due to structural damage in the myocardium. The heart’s mechanical integrity relies on a collagen-rich ECM that supports fibroblasts and myocytes. The ECM displays a significant hierarchical structure, demonstrating key organizational levels across various scales (Figure 3). This matrix, crucial for tissue repair and signaling, consists of three layers: the endocardium, myocardium, and epicardium, each about 100 µm thick. The endocardium and epicardium are composed of collagen and elastin, while the myocardium, the heart’s functional layer, contains aligned cardiomyocytes, making up about 70% of its volume (Tadevosyan et al., 2021).

Cardiomyocytes are responsible for generating electrical signals that synchronize the heart’s contractions, ensuring effective blood circulation (Holzapfel and Ogden, 2009). These cells are embedded in the ECM, which organizes them into 3D structures and facilitates electrical conduction (Vunjak-Novakovic et al., 2010). The ECM also supports a capillary network essential for delivering nutrients to the high-metabolism myocardium. Pore sizes in the ECM range from 10 to 50 μm, providing spaces for myocytes and coronary microvessels.

The heart exhibits poromechanical properties due to fluid movement through its porous ECM and the ECM’s viscoelasticity. During heart failure, pathological ECM remodeling, marked by fibrosis and collagen buildup, stiffens the myocardial tissue and disrupting heart function. The dynamic role of the ECM in cell signaling and tissue structure highlights its potential as a target for therapies aimed at reversing adverse remodeling. Understanding these processes is critical for advancing treatments, such as regenerative medicine and engineered scaffolds to restore heart function.

### 5.2 Recent advances in mimicking cardiac tissue

Myocardial infarction, characterized by diminished blood flow to the myocardium, affects millions of individuals annually and leads to a high mortality rate. The urgent need to regenerate infarcted heart tissue, which relies heavily on successful vascularization post-implantation, drives the search for effective medical interventions. Myocardial regeneration remains a focal point in contemporary research, with efforts primarily directed towards the regeneration of cardiomyocytes. The principal challenge in myocardial regeneration is the negligible proliferative capacity of the mature myocardium, which is terminally differentiated and exhibits a limited capacity for proliferation and regeneration following injury, prompting efforts to replace damaged myocardial tissue with cultured cardiomyocytes through cell injections or engineered sheets.

Cardiac tissue engineering (CTE) attempts to create biomaterials that emulate the ECM of the myocardium to foster cell growth and organ repair (Coulombe and Murry, 2014). Significant progress has been made in cell therapy, particularly in the transplantation of cardiomyocytes derived from human stem cells, which shows promise for heart regeneration (Jackman et al., 2015; Liu et al., 2022; Masumoto et al., 2014; Tenreiro et al., 2021; Pezhouman et al., 2023). However, these therapies face notable challenges, including low cell viability after transplantation and cell leakage from the implantation site (Vagnozzi et al., 2020; Menasché, 2018). Moreover, the injured myocardium’s hostile microenvironment, characterized by insufficient nutrient and oxygen supply due to poor vascularization, creates additional barriers to successful cell engraftment. Despite these hurdles, research continues to focus on developing new cell sources, improving the delivery of biophysical and biochemical stimuli delivery, and designing sophisticated 3D scaffolds replicating the native extracellular environment. A key goal of these efforts is to create biomaterials that foster the formation of a dense, functional microvasculature similar to that of healthy myocardium, essential for restoring damaged heart tissue (Shou et al., 2023). Engineered cardiac tissues require well-organized vascular networks to provide oxygen and nutrients to all residing cells. The human heart boasts a dense network of 2,000–3,000 capillaries/mm^2^; however, the microvascular density of present-day implantable heart constructs is approximately 7-fold lower than that of the natural heart (Liu et al., 2022). This discrepancy underscores the urgent necessity for therapeutic angiogenesis and creation of cardiac tissues that promote angiogenesis through *in vitro* methods.

In CTE, scaffold porosity and pore size are critical parameters influencing cell growth, tissue formation, and vascularization. Some studies report that for optimal cardiac tissue regeneration, the porosity of a scaffold should be around 90%–96% to allow for a sufficient cell migration, nutrient diffusion, and oxygen supply (Sadeghi et al., 2019; Tamimi et al., 2020). High cell density and cell-to-cell communication are also one of crucial requirements for establishing efficient electrical coupling and signal transduction in cardiac tissue. However, different pore sizes have been found to optimize various regenerative processes. For example, Pham et al. (2006), Fang et al. (2022), Navaei et al. (2016) experimentally identified 10 μm pores as a minimally critical value for efficient cellular infiltration, proliferation, and cardiac tissue remodeling, although the average diameters of cardiomyocytes, endothelial cells, and fibroblasts are usually bigger than the reported values (10–25 μm diameter). Liu et al. (2017), Yang et al. (2019b) showed that microfibrous matrices with pores of ca. 25 μm facilitated the alignment of cells into multicellular bundles and supported unimpeded cellular infiltration into scaffold layers and enhanced the structural integrity, beneficial for preventing blood vessel rupture. Thomson et al. (2013) demonstrated a high-density microtemplated fibrin scaffold with 60 μm parallel microchannels and an interconnected porous network of 27 μm pores to enhance cell retention and integration with host tissue. Seeded with cardiomyocytes, endothelial cells, and fibroblasts, the scaffold mimicked the structure and composition of native cardiac tissue. *In vitro*, results demonstrated excellent cell seeding density, survival, organization, and significant ECM deposition. Notably, endothelial cell-lined lumens formed within the scaffold channels, indicating successful revascularization. Scaffold stiffness increased by approximately 210% over 10 days. Although the authors did not provide quantitative cell seeding efficiency, fluorescent microscopy images showed a densely populated scaffold matrix. These findings align with Madden et al. (2010), who identified 60 μm pores as optimal for cell penetration and retention, while smaller interconnected pores were found to minimize the immune response by reducing fibrous capsule formation in myocardial implants (Baei et al., 2016).

It was claimed that pores with a diameter bigger than the diameter of the endothelial cells could be problematic for vascularization since endothelial cells cannot bridge the pores greater than the cell diameter (Chen et al., 2008; Zimmermann et al., 2006). However, multiple studies suggested that scaffolds with larger pores provide a better balance between cell interconnectivity and nutrient diffusion. For example, using a melt electrospinning writing, Castilho et al. (2017) fabricated a scaffold from hydroxyl-functionalized polyester and (poly(hydroxymethylglycolide-co-ε-caprolactone) blend, with pores in the range of 100–300 μm, mounted on collagen hydrogel, and reported efficient alignment of cells with directionality along fibers (4–7 µm) and significant improvement of cellular response to mechanical anisotropy. It should be noted that the reported pore sizes refer to the fabricated open channels. In contrast, the interconnected porosity of the material is demonstrated as a misalignment of the fibers, creating openings between the scaffold walls that divide its sections into designated polymeric cells. Despite the demonstration of cell alignment along the microfibers, microscopic images show that most of the cells were located on the collagen pad rather than on the scaffold. Janarthanan et al. (2022) demonstrated hydrogel scaffolds fabricated from various alginate compositions and hyaluronic acid blends, supported by ionic crosslinking via Ca^2+^, with potential implementation for cardiac or muscle tissue regeneration. The fabricated scaffolds were characterized by pronounced interconnected porosity, with pore sizes of ca. 180 μm. The authors demonstrated *in vitro* and *in vivo* cytocompatibility (from ca. 70%–100%) of the scaffolds, efficient attachment of cells, proliferation, and enhanced angiogenesis after 4 weeks. Similarly, Martins et al. (2014) fabricated chitosan/carbon conductive scaffolds for cardiac tissue applications with pore sizes ranging from 120 to 150 μm, demonstrating that these scaffolds supported neonatal rat myocyte attachment and proliferation while enhancing cardiogenic properties, including a 1.2 to 5-fold increase in the expression of cardiac genes and elevated production of the cardiac proteins Tnnc1 and Cx43, all without the need for exogenous electrical stimulation. Likewise, Bahrami et al. (2019) reported graphene foam scaffolds with pore sizes between 100 and 300 µm were effective for myocytes seeding, proliferation, ECM deposition, and enhanced vascularization.

Table 3 summarizes the selected studies on the advances in cardiac tissue engineering, focusing on the significance of the pore sizes.

**TABLE 3:** Summary of representative studies on cardiac tissue generation within porous scaffolds**.**

Cell type	Preparing method	Materials	Pore size	Results	Ref.
hBMSCs	Sonication, freeze-drying	Alginate, chitosan	50–226 µm	The scaffold showed enhanced tensile strength, cell proliferation and penetration inside the scaffold, and higher secretion of cardiac marker	Shou et al. (2023)
Rat marrow stromal cell	Elecrtrospinning	Poly(epsilon-caprolactone)	10–45 µm	Nanofibers mimicked native ECM, while microfibers supported cell migration, and cells in the scaffold proliferated, differentiated, and infiltrated through the pores	Sadeghi et al. (2019)
*In vivo* host cells	Elecrtrospinning	Thermoplastic poly(ether urethane), poly(ε-caprolactone)	11.80 ± 0.85 μm	The scaffold demonstrated improved mechanical strength, puncture resistance, anti-thrombosis ability, cell infiltration, and neotissue formation without calcification	Tamimi et al. (2020)
Neonatal rat ventricular cardiomyocytes	Lyophilization, UV light	GelMA-GNR	8–12 µm	The nanorod-incorporated hydrogels showed superior electrical and mechanical properties when compared to pure hydrogels	Pham et al. (2006)
HUVECs, CMs, CFs	Electrospinning	Poly(ethylene glycol)-poly(DL-lactide) (PELA)	25.6 ± 6.4 µm	The scaffold exhibited better mechanical properties, enhanced cell viability, and abundant capillary-like network formation with spontaneous beating rates similar to those observed in adult or neonatal rats	Fang et al. (2022)
*In vivo* host cells	Electrospinning	PCL, decellularized vascular graft		The scaffold possessed enhanced mechanical properties, supported sustained drug release, and mitigated neo-intimal hyperplasia	Navaei et al. (2016)
Rat ventricular cardiomyocytes, cardiac fibroblasts, aortic endothelial cells	Sintering	Polycarbonate, PMMA	∼27 µm	The scaffold replicated the structure and composition of native cardiac tissue and enhanced cell retention and integration with host tissue with significant ECM deposition and vascular tissue regeneration	Liu et al. (2017)
Human cardiomyocytes	Microtemplating	Poly(2-hydroxyethyl methacrylate-co-methacrylic acid	∼60 µm	The larger pore size allowed for cell penetration and retention, while smaller pores prevented immune response and supported angiogenesis	Yang et al. (2019b)
Mesenchymal stem cells (MSCs)	Heat-induced gelation	CS-GNP	25 µm	The scaffold possessed the improved electrical conductivity, structural integrity, and biomechanical compatibility with native myocardium facilitating cell survival and proliferation	Thomson et al. (2013)
Cardiac progenitor cells (CPCs)	Melt electrospinning writing	pHMGCL, PCL	150 µm	The scaffold closely mimicked the mechanical properties of native myocardial tissue and facilitated cell alignment along the scaffold’s long axis	Chen et al. (2008)
MC3T3, C20A4 cells	3D printing, freeze-drying	Alginate, hyaluronic acid	113–270 µm	The scaffold showed cytocompatibility and high cell viability, biocompatibility, increased angiogenesis, and reduced macrophage infiltration	Zimmermann et al. (2006)
Neonatal rat heart cells	Freeze-drying	Chitosan, carbon nanofibers	120–150 µm	Carbon nanofibers within the scaffold enhanced the transmission of electrical signals between the cells, myocardial cell attachment and proliferation	Castilho et al. (2017)
HUVECs, neonatal rat cardiomyocytes	Chemical vapor deposition	Nanostructured graphene foams	100–300 um	The scaffolds were biocompatible and provided an electroactive environment suitable for cardiomyocyte adhesion and proliferation, ECM deposition, and improved vascularization	Janarthanan et al. (2022)

### 5.3 Critical evaluation: the optimal pore size for CTE

In CTE, scaffold pore size plays a critical role in determining the success of myocardial regeneration by influencing cellular behavior, tissue formation, and vascularization. Smaller pores, such as those around 10–25 μm, have been shown to support efficient cellular infiltration, proliferation, and cardiac tissue remodeling. Scaffolds with pores of ca. 25 μm facilitate cell alignment into multicellular bundles, enhancing structural integrity, while 60 µm pores have been identified as optimal for cell seeding, retention, vascularization, and unimpeded transport of nutrients. Despite the claim that pores exceeding the size of endothelial cells (>25 µm) can hinder vascularization, as endothelial cells struggle to bridge larger gaps, scaffolds with larger pores (100–300 µm) were also demonstrated as an efficient morphology that offers excellent nutrient diffusion and cell interconnectivity. However, such pore sizes do not enhance cell attachment or tissue formation. Notably, scaffolds with large pore sizes exhibited significant surface roughness and contained hydrophobic and conductive materials, e.g., carbon or graphene, which compensated for the large pores and promoted efficient cell adhesion and cell-to-cell communication. In contrast, smaller pores in the range of 25–60 µm seem to strike a better balance between supporting cell attachment, enhancing vascularization, and promoting tissue regeneration, without the need for compensatory dopants, as is the case with larger pore diameters, as demonstrated across various materials in both *in vitro* and *in vivo* experiments. These pore sizes were also shown to reduce immune responses by minimizing fibrous capsule formation and macrophage infiltration, making them more suitable for cardiac tissue applications. Therefore, scaffolds with moderate pore sizes around 25–60 µm seem to be optimal for myocardial regeneration, offering both effective cell integration and sufficient nutrient and gas diffusion into the scaffold, which promotes a functional tissue formation without the need for a high surface roughness or levels of hydrophobicity, and eliminating possible drawbacks associated with the large pores.

## 6 Pore size in lung tissue engineering

### 6.1 Porous anatomical features of lung tissue

**FIGURE 4:**
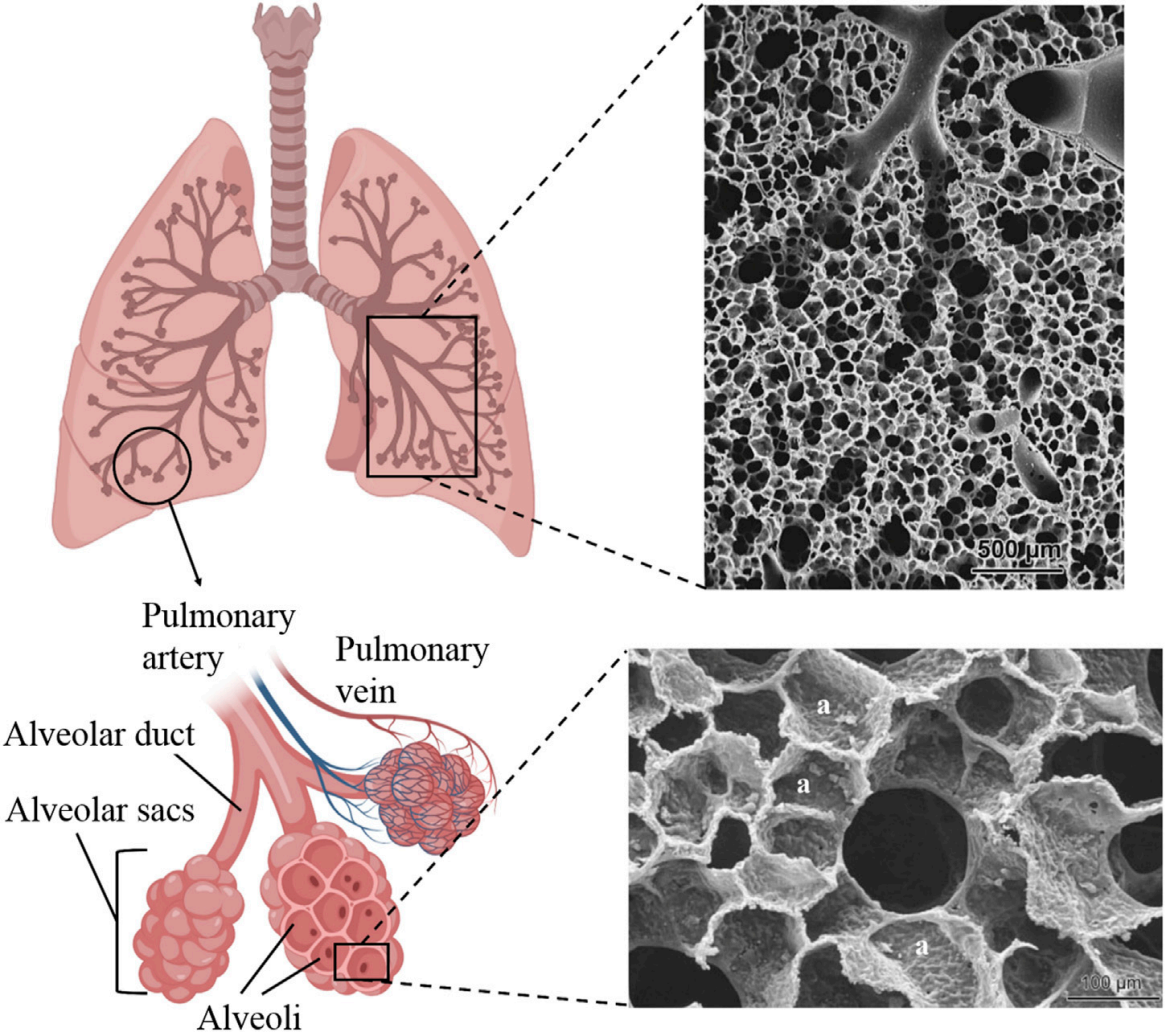
Schematic of lung tissue with branching of the airways into alveolar ducts and alveoli and SEM images of rabbit (top) and human (bottom) alveolar ducts. The SEM images were reproduced with permission from Weibel (2009), Copyright 2009, EMH Swiss Medical Publishers.

The respiratory system consists of airways and lung parenchyma. The airways include the bronchus, branching from the trachea, subdividing into bronchioles, and eventually leading to alveoli (Chaudhry and Bordoni, 2023). The lung parenchyma refers to the functional tissue of the lung involved in gas exchange, including the alveoli, alveolar ducts, and bronchioles. It has a sponge-like structure composed of bronchioles, alveoli, capillary blood vessels, and enclosed air. The alveoli, with a diameter of 200 μm, had a 3D honeycomb architecture and were the primary structural and functional units of the respiratory system, providing a large surface area for gas exchange (Figure 4).

In the lung’s area designated for gas exchange (the alveolar-capillary barrier, with a thickness of about 1 μm), a micro-scale membrane (ca. 0.1 μm thick) composed of fine collagen and elastin fibrils, along with infrequently present fibroblasts, acts as a separator between alveolar epithelial cells (facing the air) and capillary endothelial cells (facing the blood) (Weibel, 1970; Weibel, 2009). This membrane exhibits remarkable physicomechanical characteristics, such as exceptional stretchability and permeability, while preserving its structural integrity, which is crucial for the fragile structure of alveolar sacs. Basement membranes serve as permeable barriers featuring small minute pores. There is a lack of consensus regarding the precise permeability of this barrier, with estimates for smaller pores ranging from 0.5 to 2.5 nm and larger pores reaching 400 nm (Hermans and Bernard, 1998).

Furthermore, the lung parenchyma has a highly vascularized structure. The extensive network of capillaries surrounding each alveolus ensures that the blood is exposed to a large surface area, maximizing gas exchange efficiency. In most mammals, the ratio of capillary surface area to alveolar surface area is typically just below 1, ranging from 0.75 to 0.95 across different species (Gehr and Erni, 1980; Townsley, 2012). This indicates that the extensive capillary network covers a slightly smaller area than the alveoli.

### 6.2 Recent advances in mimicking lung tissue

The lung presents a formidable challenge in tissue engineering because of its intricate 3D architecture and the interconnectedness of its alveolar units with the airways and pulmonary circulation. Successful engineering of a lung requires a multifaceted matrix that not only nurtures the growth of endothelial, epithelial, and mesenchymal cells but also possesses the resilience to resist compression, the capability to facilitate efficient gas exchange, and the sophistication of developing a comprehensive vascular network. In contrast to other tissues, such as bone, cartilage, muscle, or skin, perceived as primarily solid, the alveolar lung tissue is conceptualized as a highly porous, sponge-like structure (Andrade et al., 2007; Shakir et al., 2022). The fabrication of highly porous scaffolds with appropriate mechanical characteristics has shown considerable promise over the past few decades, allowing for the replication of at least one aspect of lung tissue by effectively mimicking its highly vascular nature and stimulating blood circulation. As in other tissues, the strategy of tissue morphology mimicking underscores the necessity of a porous scaffold that can support complex functions of the lung, including the paramount task of gas exchange within the lung parenchyma.

Building on this conceptual foundation, recent studies have made significant strides in scaffold development to mimic the physical properties of lung tissue, emphasizing the critical role of pore size in facilitating lung tissue formation. This section discusses research works dedicated to developing variable scaffold designs for lung tissue engineering (LTE), including alveolar and airway regions, highlighting the role of pore dimensions in enhancing tissue regeneration.

Wang et al. (2020) developed a 3D collagen scaffold fabricated via freeze-drying of the molded construct and crosslinking using EDC/NHS, featuring a pore diameter of about 52 ± 18 μm and porosity of about 80% ± 3.6%, closely mimicking the diameter (ca. 55 ± 18 μm) and porosity (ca. 70% ± 3.6%) of natural lung tissue. The fabricated porous scaffold facilitated cell migration and provided space for their dense agglomeration, leading to the formation of capillaries along the pore walls and alveoli-like structures. Using a similar approach, O’Leary et al. (2016) fabricated a bilayer scaffold consisting of a highly porous collagen/hyaluronate matrix and nonporous film to create an epithelial barrier and efficient co-culturing of fibroblasts and bronchial epithelium. The mean pore diameter of the porous layer was ca. 70–80 μm, with porosity >98%. The authors reported successful attachment, differentiation, segregated growth of respiratory cell lines and epithelial fibroblasts, and epithelial monolayer formation. The porous structure promotes angiogenesis and guides capillary growth into separate pores, preventing disorganization. Both studies indicated that the fabricated scaffolds have comparable mechanical and structural characteristics to natural tissue. Bhowmick et al. (2018) reported successful resembling of airway epithelium, with high cell viability and enhanced marker protein expression using chitosan/collagen scaffold with a mean pore size of ca. 90 ± 7.0 μm and the porosity of ca. 80%. Due to the specific objectives of this study, no further results or characterizations, e.g., mechanical properties, ECM deposition, or angiogenesis, were provided.

Similarly, Sun et al. (2020) developed a cell-laden (embedded with live cells), gelatin-based microbubble scaffold with uniformly distributed porosity, with a mean pore diameter of ca. 110 μm (with smallest pores of ca. 80 μm) for resembling the lung alveoli in structure and mechanical properties for lung cancer treatment. The results indicated uniform cell distribution on the scaffold, excellent biocompatibility (when anti-cancer drug gemcitabine was excluded), and biological activity of the cells. However, no further evaluation of ECM formation or angiogenesis was reported due to the different focus of this work, which aimed at creating a lung tissue model for studying the potential of lung cancer drugs. Aiming to resemble alveolar ECM, Azimi et al. (2020) fabricated electrospun poly(vinylidene fluoride-co-trifluoroethylene)-based fiber mesh scaffolds using variable fabrication settings and antibacterial doping of ZnO. The obtained scaffolds exhibited an extended range of pore diameters, with ca. 80% of pores being in the sub-5 μm range, ca. 15% in the range from 10–20 μm, and a minor fraction between 20–100 μm. Due to the prevalence of microporosity, the scaffold demonstrated enhanced epithelial cell adhesion, proliferation, and significant biological activity, indicating the deposition of natural ECM. The scaffolds demonstrated minimal cytotoxicity and were found to be suitable for microvasculature infiltration, suggesting their potential to minimize the foreign body reaction due to pores that are small enough yet exceed the minimum capillary size and are compatible with red blood cell size. However, the authors indicate that the studied material demonstrates a prolonged degradation rate, which hinders the implementation of the scaffold in real *in vivo* experiments.

It should be noted that the trachea is fairly mentioned as part of lung tissue, yet the function and properties of cartilaginous tracheal and soft, highly porous alveolar tissues differ dramatically. Here, we discuss the progress made in fabricating the airway region.

Park et al. (2018a) demonstrated that omentum-cultured PCL scaffolds, fabricated via 3D printing with average pore sizes of approximately 70–100 μm, effectively supported the formation of new connective tissue and microcapillaries, facilitating revascularization and tracheal tissue regeneration. The authors noted that the pores were crucial for promoting rapid re-epithelialization, which is essential for preventing complications such as granulation tissue formation and stenosis post-implantation. In another study, the same research group (Park et al., 2018b) utilized a 4-axis 3D printing technique to fabricate a PCL-based tracheal scaffold. This technique incorporates an additional rotational axis, allowing for improved control of fiber deposition and, consequently, enhanced printing accuracy. This resulted in the creation of uniform square-shaped pores measuring 100 × 100 μm and a higher total porosity (up to 40%) compared to the traditional 3D printing approach, which achieved approximately 30% porosity. The authors demonstrated that consistent and uniform porosity, along with interconnectivity, significantly contributes to the mechanical resilience of the scaffolds and ensures better cell ingrowth into the porous regions, effectively mimicking the natural trachea. Gao et al. (2022) prepared a highly porous (porosity >92%) ring-shaped silk fibroin-reinforced decellularized cartilaginous matrix scaffold with an average pore diameter of ca. 200 μm for the fabrication of trachea tissue regeneration. In this work, the authors highlight the role of silk fibroin in contraction resistance capacity and the slow degradation rate of the scaffold. A comprehensive set of *in vitro* and *in vivo* data showed that the scaffolds possess negligible cytotoxicity and provide an excellent matrix for cell infiltration, attachment, proliferation, and chondrogenic capacity, successfully demonstrated in the rabbit model during segmental tracheal defect treatment. Similarly, Xu et al. (2021) achieved extensive tracheal reconstruction using chondroitin-sulfate-incorporating type-II atelocollagen ring-shaped scaffolds with pore size distribution ranging from ca. 50–100 µm. The authors also compared scaffolds of other compositions, e.g., collagen-I and collagen-II scaffolds, which are characterized by larger pore diameters (25–150 μm and 150–250 μm, respectively). The latter compositions revealed different bioresponses on the cellular level, thus the outcomes were not directly comparable due to differences in scaffold composition. Dye et al. (2020), investigated the effect of polymer type, degradation rate, and pore interconnectivity of the scaffolds on the transplantation efficiency for airway tissue regeneration. Amid conclusions, the authors found that higher interconnectivity of pores ranging from 10–100 μm promotes better fusion of lung organoids into airway-like structures, while smaller pores or less interconnected scaffolds led to less organized or smaller tissue formations.

Table 4 summarizes the selected publications on the advances in lung tissue engineering, focusing on the significance of the pore sizes.

**TABLE 4:** Summary of representative studies on lung tissue generation within porous scaffolds.

Tissue type	Cell type	Preparing method	Materials	Pore size	Results	Ref.
Lung	Human lung cancer cells A549, rat alveolar type two epithelial cell line	Freeze-drying	Collagen	52.67 ± 18.39 μm	The implanted scaffold supported cell infiltration, formation of micro-vessels and alveolar-like structures, and lung regeneration	Andrade et al. (2007)
Tracheobronchial region	Calu-3 bronchial epithelium cells, Wi38 human embryonic lung fibroblasts	Evaporation, freeze-drying	Collagen-hyaluronate	70–80 μm	The cells differentiated, accompanied by elevated mucin production, greater ciliation, and the development of tight intercellular junctions forming a submucosal tissue-like structure	Shakir et al. (2022)
Lower airway epithelium	HSAEpCs	Phase-separation, freeze-drying	Chitosan-collagen	Major pores of 91.61 ± 7.64 μm, minor pores of 54.33 ± 7.11 μm	The scaffold facilitated the formation of a uniform cell layer that closely mimics the *in vivo* small airway epithelium, acting as a barrier between the circulation and the external environment	Wang et al. (2020)
Lung alveoli	A549 cloned with luciferase gene	Microfluidic channel, gas foaming	Gelatin	∼113.62 μm	The scaffold possessed favorable biocompatibility and cellular response and mimicked the structure of lung alveoli	O’Leary et al. (2016)
Lung alveoli	A549 lung cancer cells	Electrospinning	P(VDF-TrFE)	<5 µm	The scaffold supported cell growth, showed adequate mechanical strength and piezoelectricity, and exhibited immunomodulatory and antibacterial properties	Bhowmick et al. (2018)
Trachea	No cells. Cultivation in omentum	3D bioprinting	PCL	0.0043 mm^2^ superior ∼0.0049 mm^2^ inferior ∼0.0058 mm^2^ anterior ∼0.0162 mm^2^ inner	The scaffold demonstrated rapid cell infiltration and formation of the highly organized tissue consisting of ciliated respiratory epithelium and submucosal part	Sun et al. (2020)
Trachea	NIH3T3	3D bioprinting	PCL	-	The scaffold integrated well with host tissue and showed improved mechanical properties, cell ingrowth, and formation of new epithelium with neocartilage regeneration	Azimi et al. (2020)
Trachea	BMSCs	Freeze-drying	Silk fibroin, decellularized cartilaginous matrix	∼206.7 ± 12.5 μm	The scaffold showed negligible cytotoxicity and provided an excellent matrix for cell infiltration, attachment, proliferation, and chondrogenic capacity	Park et al. (2018a)
Trachea	BMSCs	Freeze-drying	Collagen	ca. 50–100 µm	The scaffold demonstrated enhanced chondrogenic capacity, rigidity of the lumen, flexibility along its length, and sufficient vascular perfusion	Park et al. (2018b)
Lung organoids	Human embryonic stem cells	Gas foaming, particulate leaching	PLG, PCL, PEG	10–100 μm	The organoids replicated both the architecture and cellular diversity of a mature airway attributed to pore interconnectivity and polymer degradation	Gao et al. (2022)

### 6.3 Critical evaluation: the optimal pore size for LTE

Based on the reviewed literature on lung tissue regeneration, relatively few studies report porosity characteristics and pore dimensions or examine their impact on biological responses and tissue formation efficiency. However, some conclusions can still be drawn. Unlike other tissue types, alveolar tissue regeneration uses scaffolds with more homogeneous pore sizes, typically between 50 and 100 μm. These pore sizes are frequently reported to facilitate cell migration, capillary formation, and native tissue restructuring while maintaining suitable mechanical properties such as elasticity and structural stability. Scaffolds with smaller pores, below 5 μm, as reported by Azimi et al. (2020), have been shown to enhance cell adhesion and seeding efficiency, which aligns with general findings in other studies discussed earlier. However, a significant fraction of pores in the 20–100 μm range suggests that pores within this size range are crucial for successful tissue formation and adequate vascularization. Therefore, to mimic alveolar tissue effectively, scaffolds with highly interconnected porosity and pore sizes ranging from a few to tens of micrometers are considered optimal, as they maximize cell adhesion, ensure efficient nutrient flow, and provide sufficient space for vascularization.

For trachea-mimicking tissue, higher variability of pore sizes is presented, ranging from 10 to 200 μm, and reported as suitable for epithelial cell attachment, differentiation, and revascularization. While trying to evaluate the necessity or favorable effects of different pore sizes, taking into account generally considered aspects of cell adhesion, cell-to-cell communication, nutrition/waste diffusion, and vascularization, we did not see significant advantages of using pores >100 μm, as Dye et al. (2020) demonstrated. Conversely, pores <10 μm in diameter should also be avoided to maximize the efficiency of tissue formation, ideally sticking to the middle range pores used, >50 μm.

## 7 Discussion and future directions

The literature shows no consensus on the ideal pore size and scaffold morphology, with a wide range of reported pore sizes. Tissue formation and associated biological processes are complex and cannot be reduced to a single factor. Several scaffold features contribute to the outcomes, including mechanical properties, morphology, material composition, roughness, porosity, pore interconnectivity, and degradation characteristics. Scaffolds are used to cultivate various tissue types, and many have regions with different mechanical and morphological properties within the same tissue. This complexity makes analyzing scaffold-cell interactions more challenging.

Mimicking the natural morphology of tissues is widely considered the most promising approach. Scaffolds with varied designs - from simple structures with uniform morphology to advanced, multilayered designs with different pore sizes and gradients - promote biological responses and improve tissue formation efficiency. However, this mimicking is not always fully implemented, likely due to challenges in balancing the bioinductive and cytocompatible properties of the materials with their physical characteristics. These limitations often lead to compromises in scaffold design and morphology. Additionally, fabrication methods and chemical processes, such as UV crosslinking or cryogelation, can affect the scaffold’s porosity and pore sizes, deviating from the initial design. Thus, it is crucial to carefully evaluate the reported results and terminology, as significant variability exists across studies. Scaffolds intended for the same tissue type can exhibit pore sizes that differ by orders of magnitude.

For example, lung tissue scaffolds favor uniform pore sizes in the alveolar and airway regions. In contrast, bone, cartilage, and skin tissue engineering pore sizes range from sub-micron to over 400 µm. Despite this variation, successful tissue development has been reported in many studies. This suggests that the relationship between scaffold properties and tissue growth is multifaceted and dynamic. Key physiological processes standard to all tissues, such as cell adhesion, infiltration, cell-to-cell communication, nutrient and waste transport, gas diffusion, ECM deposition, and vascularization are influenced by scaffold pore size and porosity (Loh and Choong, 2013; Xia and Luo, 2022; Murphy et al., 2010).

Several general observations from the reviewed studies can be highlighted:

a) Cells adhere better to scaffolds with high surface roughness and interconnected pores similar in size to the cells.
b) Smaller pores enhance cell seeding efficiency, increase cell density, and promote cell-to-cell communication.
c) Cell infiltration and the transport of nutrients, growth factors, and gases are limited when pore sizes are smaller than the diffusing substances.
d) Incorporating larger channels can improve mass transport and vascularization in scaffolds with interconnected pores smaller than 100 µm.
e) Pores larger than 100 µm generally support vascularization.
f) In hydrogels and polymer-based scaffolds, porosity, and pore size significantly affect scaffold stability and degradation rate.

Despite using scaffolds with suboptimal pore sizes (either too small for cell infiltration or too large for effective nutrient diffusion), some studies have reported outcomes comparable to those achieved with “optimal” pore sizes. This observation leads to two key conclusions: first, porosity and pore size are dynamic properties, often influenced by the material’s swelling behavior in physiological conditions. Materials with high swelling capacity can reveal much smaller pores than observed during initial characterization. Second, pore size and degradation rate are interconnected (Molina et al., 2021). In hydrogels, larger pores accelerate degradation due to a higher surface area-to-volume ratio, which enhances hydrolysis. While large pores may lead to faster scaffold breakdown, they can also increase in size over time, making them more conducive to vascularization.

Based on these insights, three key takeaways can be summarized:

1. Gradients and Layering: Multilayered and gradient scaffold designs that incorporate regions with both small and large pores consistently outperform scaffolds with uniform pore sizes. This strategy promotes cell attachment, migration, and vascularization more effectively by mimicking natural tissue structures.
2. Size Ranges: Smaller pores (<100 µm) enhance cell attachment and early tissue formation, while larger pores (100–400 µm) support nutrient transport, vascularization, and long-term tissue stability. However, excessively large pores (>400 µm) offer limited benefits and may weaken scaffold mechanical properties.
3. Material and Morphology Interaction: The relationship between scaffold material properties (roughness, hydrophobicity, and degradation rate) and pore size is crucial. These material properties can compensate for non-ideal pore sizes and significantly affect scaffold performance.

While this review provides recommendations for scaffold pore dimensions in tissue engineering, it is essential to recognize that porosity and pore size change over time during tissue cultivation. Scaffold designs must be tailored to the target tissue’s specific biological and mechanical needs. Advanced designs, such as gradients in pore size or material composition, should be optimized to support each phase of tissue regeneration (Karageorgiou and Kaplan, 2005; Tavakoli et al., 2023; Di Luca et al., 2016).

Future research in scaffold development should focus on several key areas. First, advanced fabrication techniques should be prioritized to improve control over scaffold morphology, particularly in creating gradient and multilayered designs that mimic natural tissues. Techniques like 3D printing, bioprinting, and hybrid manufacturing methods can allow for precise adjustments in pore size distribution and material properties, enhancing tissue integration and function (Adel et al., 2022; Kalogeropoulou et al., 2024; Koyyada and Orsu, 2021). Second, more research is needed to understand the dynamic changes in scaffold properties, such as pore size and interconnectivity during degradation. Developing scaffolds that adapt over time could optimize nutrient diffusion and vascularization during different stages of tissue growth. Third, material characteristics like surface roughness, hydrophobicity, and degradation rate must be systematically explored with pore size. These factors can be tailored to improve scaffold performance, even when pore sizes are suboptimal. Lastly, scaffold designs should be further refined based on the specific needs of different tissue types. Bone, cartilage, cardiac, and lung tissues have unique requirements, and scaffold properties must be tailored to ensure long-term tissue stability, integration, and vascularization. Future research can significantly improve outcomes in tissue engineering by advancing scaffold designs to mimic the dynamic nature of biological tissues better.

## 8 Conclusion

This review systematically examines the role of scaffold pore size and porosity across various fields of tissue engineering, including skin, bone, cardiovascular, and lung tissue regeneration. The literature reviewed highlights the critical influence of pore characteristics in replicating the native microenvironment essential for optimal cell function and integration. Specifically, scaffold porosity is discussed in relation to key processes such as cellular interactions, migration, adhesion, proliferation, and differentiation, which are crucial for successful tissue regeneration. Although the optimal pore size varies across tissues, some general conclusions can be drawn, reflecting the complex interplay between scaffold properties and biological needs. The studies underscore the importance of tailoring scaffold design, with pore size and structure being central to tissue regeneration. However, this review focuses solely on the aspect of porosity and should not be viewed as a comprehensive guide for scaffold selection in tissue engineering applications.

## References

[B1] Abbasi N. Hamlet S. Love R. M. Nguyen N. (2020). Porous scaffolds for bone regeneration. J. Sci. Adv. Mater. Devices 5 (1), 1–9. 10.1016/j.jsamd.2020.01.007

[B2] Adel I. M. ElMeligy M. F. Elkasabgy N. A. (2022). Conventional and recent trends of scaffolds fabrication: a superior mode for tissue engineering. Pharmaceutics 14 (2), 306. 10.3390/pharmaceutics14020306 35214038 PMC8877304

[B3] Akay G. Birch M. A. Bokhari M. A. (2004). Microcellular polyHIPE polymer supports osteoblast growth and bone formation *in vitro* . Biomaterials 25 (18), 3991–4000. 10.1016/j.biomaterials.2003.10.086 15046889

[B4] Akilbekova D. Boddupalli A. Bratlie K. M. (2018). The effect of polarized light on the organization of collagen secreted by fibroblasts. Lasers Med. Sci. 33, 539–547. 10.1007/s10103-017-2398-0 29192340

[B5] Akilbekova D. Mektepbayeva D. (2017). “Patient specific *in situ* 3D printing,” in 3D Printing in medicine. Editor Kalaskar D. M. (Cambridge: Woodhead Publishing), 91–113. 10.1016/B978-0-08-100717-4.00004-1

[B6] Akilbekova D. Turlybekuly A. (2023). “Patient-specific 3D bioprinting for *in situ* tissue engineering and regenerative medicine,” in 3D Printing in medicine. Editor Kalaskar D. M. (Cambridge: Woodhead Publishing), 149–178. 10.1016/B978-0-323-89831-7.00003-1

[B7] Alkentar R. Kladovasilakis N. Tzetzis D. Mankovits T. (2022). Effects of pore size parameters of titanium additively manufactured lattice structures on the osseointegration process in orthopedic applications: a comprehensive review. Crystals 13 (1), 113. 10.3390/cryst13010113

[B8] Almela T. Brook I. Khoshroo K. Rasoulianboroujeni M. Fahimipour F. Tahriri M. (2017). Simulation of cortico-cancellous bone structure by 3D printing of bilayer calcium phosphate-based scaffolds. Bioprinting 6, 1–7. 10.1016/j.bprint.2017.04.001

[B9] Andrade C. F. Wong A. P. Waddell T. K. Keshavjee S. Liu M. (2007). Cell-based tissue engineering for lung regeneration. Am. J. Physiology-lung Cell. Mol. Physiology 292 (2), L510–L518. 10.1152/ajplung.00175.2006 17028264

[B10] Azimi B. Bafqi M. S. S. Fusco A. Ricci C. Gallone G. Bagherzadeh R. (2020). ElectroSpun ZNO/POly(Vinylidene Fluoride-Trifluoroethylene) scaffolds for lung tissue engineering. Tissue Eng. Part A 26 (23–24), 1312–1331. 10.1089/ten.tea.2020.0172 32842903

[B11] Baei P. Jalili-Firoozinezhad S. Rajabi-Zeleti S. Tafazzoli-Shadpour M. Baharvand H. Aghdami N. (2016). Electrically conductive gold nanoparticle-chitosan thermosensitive hydrogels for cardiac tissue engineering. C, Mater. Biol. Appl. 63, 131–141. 10.1016/j.msec.2016.02.056 27040204

[B12] Bahrami S. Baheiraei N. Mohseni M. Razavi M. Ghaderi A. Azizi B. (2019). Three-dimensional graphene foam as a conductive scaffold for cardiac tissue engineering. J. Biomaterials Appl. 34 (1), 74–85. 10.1177/0885328219839037 30961432

[B13] Bartoš M. Suchý T. Foltán R. (2018). Note on the use of different approaches to determine the pore sizes of tissue engineering scaffolds: what do we measure? Biomed. Eng. OnLine 17, 110. 10.1186/s12938-018-0543-z 30119672 PMC6098612

[B14] Bhowmick R. Derakhshan T. Liang Y. Ritchey J. W. Liu L. Gappa-Fahlenkamp H. (2018). A Three-Dimensional human Tissue-Engineered lung model to study influenza A infection. Tissue Eng. Part A 24 (19–20), 1468–1480. 10.1089/ten.tea.2017.0449 29732955 PMC6198767

[B15] Boddupalli A. Akilbekova D. Bratlie K. M. (2020). Poly-l-arginine modifications alter the organization and secretion of collagen in SKH1-E mice. Mater. Sci. Eng. C 106, 110143. 10.1016/j.msec.2019.110143 31753344

[B16] Bružauskaitė I. Bironaitė D. Bagdonas E. Bernotienė E. (2015). Scaffolds and cells for tissue regeneration: different scaffold pore sizes—different cell effects. Cytotechnology 68 (3), 355–369. 10.1007/s10616-015-9895-4 26091616 PMC4846637

[B17] Büyük N. İ. Aksu D. Köse G. T. (2022). Effect of different pore sizes of 3D printed PLA-based scaffold in bone tissue engineering. Int. J. Polym. Mater. 72 (13), 1021–1031. 10.1080/00914037.2022.2075869

[B18] Calore A. R. Srinivas V. Groenendijk L. Serafim A. Stancu I. C. Wilbers A. (2023). Manufacturing of scaffolds with interconnected internal open porosity and surface roughness. Acta biomater. 156, 158–176. 10.1016/j.actbio.2022.07.017 35868592

[B19] Castilho M. Feyen D. Flandes-Iparraguirre M. Hochleitner G. Groll J. Doevendans A. P. (2017). Melt electrospinning writing of poly-hydroxymethylglycolide-co -ε-Caprolactone-Based scaffolds for cardiac tissue engineering. Adv. Healthc. Mater. 6 (18), 1700311. 10.1002/adhm.201700311 PMC7116102 28699224

[B20] Cavo M. Scaglione S. (2016). Scaffold microstructure effects on functional and mechanical performance: integration of theoretical and experimental approaches for bone tissue engineering applications. Mater. Sci. Eng. C 68, 872–879. 10.1016/j.msec.2016.07.041 27524090

[B21] Chao L. Jiao C. Liang H. Xie D. Shen L. Liu Z. (2021). Analysis of mechanical properties and permeability of trabecular-like porous scaffold by additive manufacturing. Front. Bioeng. Biotechnol. 9, 779854. 10.3389/fbioe.2021.779854 34993188 PMC8724551

[B22] Chaudhry R. Bordoni B. (2023). Anatomy, thorax, lungs. StatPearls NCBI bookshelf. Available at: https://www.ncbi.nlm.nih.gov/books/NBK470197/. 29262068

[B23] Chen Q. Z. Harding S. E. Ali N. N. Lyon A. R. Boccaccini A. R. (2008). Biomaterials in cardiac tissue engineering: ten years of research survey. Mater. Sci. Eng. R Rep. 59 (1), 1–37. 10.1016/j.mser.2007.08.001

[B24] Choi W. S. Kim J. H. Ahn C. B. Lee J. H. Kim Y. J. Son K. H. (2021). Development of a multi-layer skin substitute using human hair keratinic extract-based hybrid 3D printing. Polymers 13 (16), 2584. 10.3390/polym13162584 34451127 PMC8401121

[B25] Chu B. He J. Wang Z. Liu L. Li X. Wu C.-X. (2021). Proangiogenic peptide nanofiber hydrogel/3D printed scaffold for dermal regeneration. Chem. Eng. J. 424, 128146. 10.1016/j.cej.2020.128146

[B26] Cidonio G. Cooke M. Glinka M. Dawson J. I. Grover L. Oreffo R. O. C. (2019). Printing bone in a gel: using nanocomposite bioink to print functionalised bone scaffolds. Mater. Today Bio 4, 100028. 10.1016/j.mtbio.2019.100028 PMC6894340 31853520

[B27] Cooper D. M. Thomas C. D. Clement J. G. Turinsky A. L. Sensen C. W. Hallgrímsson B. (2013). Age-dependent change in the 3D structure of cortical porosity at the human femoral midshaft. Bone 53 (2), 957–965. 10.1016/j.bone.2006.11.011 17223618

[B28] Coulombe K. L. Murry C. E. (2014). Vascular perfusion of implanted human engineered cardiac tissue. Proc. IEEE . Annu. Northeast Bioeng. Conf. 2014, 1–2. 10.1109/nebec.2014.6972763 PMC4721559 26807015

[B29] Currey J. D. (2002). Bones: structure and mechanics. USA: Princeton University Press. Available at: http://www.jstor.org/stable/j.ctt4cg9wv.

[B30] Dave K. Gomes V. G. (2019). Interactions at scaffold interfaces: effect of surface chemistry, structural attributes and bioaffinity. Mater. Sci. Eng. C 105, 110078. 10.1016/j.msec.2019.110078 31546353

[B31] Demir A. K. Elçin A. E. Elçin Y. M. (2018). Strontium-modified chitosan/montmorillonite composites as bone tissue engineering scaffold. Mater. Sci. and Eng. C. Mater. Biol. Appl. 89, 8–14. 10.1016/j.msec.2018.03.021 29752122

[B32] Di Luca A. Longoni A. Criscenti G. Mota C. van Blitterswijk C. V. Moroni L. (2016). Toward mimicking the bone structure: design of novel hierarchical scaffolds with a tailored radial porosity gradient. Biofabrication 8 (4), 045007. 10.1088/1758-5090/8/4/045007 27725338

[B33] Dye B. R. Youngblood R. L. Oakes R. S. Kasputis T. Clough D. Spence J. R. (2020). Human lung organoids develop into adult airway-like structures directed by physico-chemical biomaterial properties. Biomaterials 234, 119757. 10.1016/j.biomaterials.2020.119757 31951973 PMC6996062

[B34] Eichholz K. F. Freeman F. E. Pitacco P. Nulty J. Ahern D. Burdis R. (2022). Scaffold microarchitecture regulates angiogenesis and the regeneration of large bone defects. Biofabrication 14 (4), 045013. 10.1088/1758-5090/ac88a1 35947963

[B35] Einhorn T. A. Gerstenfeld L. C. (2015). Fracture healing: mechanisms and interventions. Nat. Rev. Rheumatol. 11 (1), 45–54. 10.1038/nrrheum.2014.164 25266456 PMC4464690

[B36] Fang Z. Xiao Y. Geng X. Jia L. Xing Y. Ye L. (2022). Fabrication of heparinized small diameter TPU/PCL Bi-layered artificial blood vessels and *in vivo* assessment in a rabbit carotid artery replacement model. Biomater. Adv. 133, 112628. 10.1016/j.msec.2021.112628 35527159

[B37] Farazin A. Zhang C. Gheisizadeh A. Shahbazi A. (2023). 3D bio-printing for use as bone replacement tissues: a review of biomedical application. Biomed. Eng. Adv. 5, 100075. 10.1016/j.bea.2023.100075

[B38] Flores-Jiménez M. S. Garcia-Gonzalez A. Fuentes-Aguilar R. Q. (2023). Review on porous scaffolds generation process: a tissue engineering approach. ACS Appl. Bio Mater. 6 (1), 1–23. 10.1021/acsabm.2c00740 36599046

[B39] Gao E. Li G. Cao R. Xia H. Xu Y. Jiang G. (2022). Bionic tracheal tissue regeneration using a ring-shaped scaffold comprised of decellularized cartilaginous matrix and silk fibroin. Compos. Part B Eng. 229, 109470. 10.1016/j.compositesb.2021.109470

[B40] Gayer C. Ritter J. Bullemer M. Grom S. Jauer L. Meiners W. (2019). Development of a solvent-free polylactide/calcium carbonate composite for selective laser sintering of bone tissue engineering scaffolds. Mater. Sci. Eng. C 101, 660–673. 10.1016/j.msec.2019.03.101 31029360

[B41] Gehr P. Erni H. (1980). Morphometric estimation of pulmonary diffusion capacity in two horse lungs. Respir. Physiol. 41 (2), 199–210. 10.1016/0034-5687(80)90052-3 7433781

[B42] Grémare A. Guduric V. Bareille R. Heroguez V. Latour S. L'heureux N. (2017). Characterization of printed PLA scaffolds for bone tissue engineering. J. Biomed. Mater Res. A 106 (4), 887–894. 10.1002/jbm.a.36289 29105943

[B43] Gu Y. Zhang J. Zhang X. Liang G. Xu T. Niu W. (2019). Three-dimensional printed Mg-doped β-TCP bone tissue engineering scaffolds: effects of magnesium ion concentration on osteogenesis and angiogenesis *in vitro* . Tissue Eng. Regen. Med. 16 (4), 415–429. 10.1007/s13770-019-00192-0 31413945 PMC6675836

[B44] Gupte M. J. Swanson W. B. Hu J. Jin X. Ma H. Zhang Z. (2018). Pore size directs bone marrow stromal cell fate and tissue regeneration in nanofibrous macroporous scaffolds by mediating vascularization. Acta Biomater. 82, 1–11. 10.1016/j.actbio.2018.10.016 30321630 PMC6258662

[B45] Henkel J. Woodruff M. A. Epari D. R. Steck R. Glatt V. Dickinson I. C. (2013). Bone regeneration based on tissue engineering conceptions - a 21st century perspective. Bone Res. 1 (3), 216–248. 10.4248/BR201303002 26273505 PMC4472104

[B46] Hermans C. Bernard A. (1998). Pneumoproteinaemia: a new perspective in the assessment of lung disorders. Eur. Respir. J. 11 (4), 801–803. 10.1183/09031936.98.11040801 9623679

[B47] Hernandez J. L. Woodrow K. A. (2022). Medical applications of porous biomaterials: features of porosity and tissue-specific implications for biocompatibility. Adv. Healthc. Mater 11 (9), e2102087. 10.1002/adhm.202102087 35137550 PMC9081257

[B48] Holzapfel G. A. Ogden R. W. (2009). Constitutive modelling of passive myocardium: a structurally based framework for material characterization. Philosophical Trans. R. Soc. A Math. Phys. Eng. Sci. 367 (1902), 3445–3475. 10.1098/rsta.2009.0091 19657007

[B49] Ishikawa K. (2010). Bone substitute fabrication based on dissolution-precipitation reactions. Materials 3 (2), 1138–1155. 10.3390/ma3021138

[B50] Jackman C. P. Shadrin I. Y. Carlson A. L. Bursac N. (2015). Human cardiac tissue engineering: from pluripotent stem cells to heart repair. Curr. Opin. Chem. Eng. 7, 57–64. 10.1016/j.coche.2014.11.004 25599018 PMC4293542

[B51] Jafari M. Paknejad Z. Rad M. R. Motamedian S. R. Eghbal M. J. Nadjmi N. (2017). Polymeric scaffolds in tissue engineering: a literature review. J. Biomed. Mater. Res. Part B Appl. biomaterials 105 (2), 431–459. 10.1002/jbm.b.33547 26496456

[B52] Janarthanan G. Kim J. H. Kim I. Lee C. Chung E. J. Noh I. (2022). Manufacturing of self-standing multi-layered 3D-bioprinted alginate-hyaluronate constructs by controlling the cross-linking mechanisms for tissue engineering applications. Biofabrication 14 (3), 035013. 10.1088/1758-5090/ac6c4c 35504259

[B53] Jia G. Huang H. Niu J. Chen C. Weng J. Yu F. (2021). Exploring the interconnectivity of biomimetic hierarchical porous Mg scaffolds for bone tissue engineering: effects of pore size distribution on mechanical properties, degradation behavior and cell migration ability. J. Magnesium Alloys 9 (6), 1954–1966. 10.1016/j.jma.2021.02.001

[B54] Jones A. C. Sheppard A. Sok R. Arns C. Limaye A. Averdunk H. (2004). Three-dimensional analysis of cortical bone structure using X-ray micro-computed tomography. Phys. A Stat. Mech. its Appl. 339 (1-2), 125–130. 10.1016/j.physa.2004.03.046

[B55] Kalogeropoulou M. Díaz-Payno P. J. Mirzaali M. J. van Osch G. J. V. M. Fratila-Apachitei L. E. Zadpoor A. A. (2024). 4D printed shape-shifting biomaterials for tissue engineering and regenerative medicine applications. Biofabrication 16 (2), 022002. 10.1088/1758-5090/ad1e6f 38224616

[B56] Karageorgiou V. Kaplan D. (2005). Porosity of 3D biomaterial scaffolds and osteogenesis. Biomaterials 26 (27), 5474–5491. 10.1016/j.biomaterials.2005.02.002 15860204

[B57] Kilic Bektas C. Kimiz I. Sendemir A. Hasirci V. Hasirci N. (2018). A bilayer scaffold prepared from collagen and carboxymethyl cellulose for skin tissue engineering applications. J. biomaterials Sci. 29 (14), 1764–1784. 10.1080/09205063.2018.1498718 29999478

[B58] Koyyada A. Orsu P. (2021). Recent advancements and associated challenges of scaffold fabrication techniques in tissue engineering applications. Regen. Eng. Transl. Med. 7, 147–159. 10.1007/s40883-020-00166-y

[B59] Kuboki Y. Takita H. Kobayashi D. Tsuruga E. Inoue M. Murata M. (1998). BMP-induced osteogenesis on the surface of hydroxyapatite with geometrically feasible and nonfeasible structures: topology of osteogenesis. J. Biomed. Mater. Res. 39 (2), 190–199. 10.1002/(sici)1097-4636(199802)39:2<190::aid-jbm4>3.0.co;2-k 9457547

[B60] Kumar V. Kumar A. Chauhan N. S. Yadav G. Goswami M. Packirisamy G. (2022). Design and fabrication of a dual protein-based trilayered nanofibrous scaffold for efficient wound healing. ACS Appl. Bio Mater. 5 (6), 2726–2740. 10.1021/acsabm.2c00200 35594572

[B61] Liu D. Nie W. Li D. Wang W. Zheng L. Zhang J. (2019). 3D printed PCL/SrHA scaffold for enhanced bone regeneration. Chem. Eng. J. 362, 269–279. 10.1016/j.cej.2019.01.015

[B62] Liu H. Liu L. Tan H. Yan G. Xue B. (2023). Definition of pore size in 3D-printed porous implants: a review. ChemBioEng Rev. 10 (2), 167–173. 10.1002/cben.202200043

[B63] Liu Y. Xu G. Wei J. Wu Q. Li X. (2017). Cardiomyocyte coculture on layered fibrous scaffolds assembled from micropatterned electrospun mats. C, Mater. Biol. Appl. 81, 500–510. 10.1016/j.msec.2017.08.042 28888004

[B64] Liu Y. Zhang Y. Mei T. Cao H. Hu Y. Jia W. (2022). hESCs-derived early vascular cell spheroids for cardiac tissue vascular engineering and myocardial infarction treatment. Adv. Sci. Weinheim, Baden-Wurttemberg, Ger. 9 (9), e2104299. 10.1002/advs.202104299 PMC8948571 35092352

[B65] Loh Q. L. Choong C. (2013). Three-dimensional scaffolds for tissue engineering applications: role of porosity and pore size. Tissue Eng. Part B Rev. 19, 485–502. 10.1089/ten.teb.2012.0437 23672709 PMC3826579

[B66] Loi F. Córdova L. A. Pajarinen J. Lin T. H. Yao Z. Goodman S. B. (2016). Inflammation, fracture and bone repair. Bone 86, 119–130. 10.1016/j.bone.2016.02.020 26946132 PMC4833637

[B67] Lutzweiler G. Ndreu Halili A. Engin Vrana N. (2020). The overview of porous, bioactive scaffolds as instructive biomaterials for tissue regeneration and their clinical translation. Pharmaceutics 12 (7), 602. 10.3390/pharmaceutics12070602 32610440 PMC7407612

[B68] Madden L. R. Mortisen D. J. Sussman E. M. Dupras S. K. Fugate J. A. Cuy J. L. (2010). Proangiogenic scaffolds as functional templates for cardiac tissue engineering. Proc. Natl. Acad. Sci. U. S. A. 107 (34), 15211–15216. 10.1073/pnas.1006442107 20696917 PMC2930533

[B69] Malda J. Klein T. J. Upton Z. (2007). The roles of hypoxia in the *in vitro* engineering of tissues. Tissue Eng. 13 (9), 2153–2162. 10.1089/ten.2006.0417 17516855

[B70] Martin J. R. Gupta M. K. Page J. M. Yu F. Davidson J. M. Guelcher S. A. (2014). A porous tissue engineering scaffold selectively degraded by cell-generated reactive oxygen species. Biomaterials 35 (12), 3766–3776. 10.1016/j.biomaterials.2014.01.026 24491510 PMC3975079

[B71] Martins A. M. Eng G. Caridade S. G. Mano J. F. Reis R. L. Vunjak-Novakovic G. (2014). Electrically conductive chitosan/carbon scaffolds for cardiac tissue engineering. Biomacromolecules 15 (2), 635–643. 10.1021/bm401679q 24417502 PMC3983145

[B72] Masumoto H. Ikuno T. Takeda M. Fukushima H. Marui A. Katayama S. (2014). Human iPS cell-engineered cardiac tissue sheets with cardiomyocytes and vascular cells for cardiac regeneration. Sci. Rep. 4, 6716. 10.1038/srep06716 25336194 PMC4205838

[B73] Menasché P. (2018). Cell therapy trials for heart regeneration - lessons learned and future directions. Nat. Rev. Cardiol. 15 (11), 659–671. 10.1038/s41569-018-0013-0 29743563

[B74] Meskinfam M. Bertoldi S. Albanese N. Cerri A. Tanzi M. C. Imani R. (2018). Polyurethane foam/nano hydroxyapatite composite as a suitable scaffold for bone tissue regeneration. Mater. Sci. Eng. C 82, 130–140. 10.1016/j.msec.2017.08.064 29025641

[B75] Molina M. I. E. Malollari K. G. Komvopoulos K. (2021). Design challenges in polymeric scaffolds for tissue engineering. Front. Bioeng. Biotechnol. 9, 617141. 10.3389/fbioe.2021.617141 34195178 PMC8236583

[B76] Mukasheva F. Moazzam M. Yernaimanova B. Shehzad A. Zhanbassynova A. Berillo D. (2024). Design and characterization of 3D printed pore gradient hydrogel scaffold for bone tissue engineering. Bioprinting 39, e00341. 10.1016/j.bprint.2024.e00341

[B77] Murphy C. M. Haugh M. G. O’Brien F. J. (2010). The effect of mean pore size on cell attachment, proliferation and migration in collagen–glycosaminoglycan scaffolds for bone tissue engineering. Biomaterials 31 (3), 461–466. 10.1016/j.biomaterials.2009.09.063 19819008

[B78] Murphy C. M. O’Brien F. J. (2010). Understanding the effect of mean pore size on cell activity in collagen-glycosaminoglycan scaffolds. Cell. Adhesion and Migr. 4 (3), 377–381. 10.4161/cam.4.3.11747 PMC2958613 20421733

[B79] Navaei A. Saini H. Christenson W. Sullivan R. T. Ros R. Nikkhah M. (2016). Gold nanorod-incorporated gelatin-based conductive hydrogels for engineering cardiac tissue constructs. Acta Biomater. 41, 133–146. 10.1016/j.actbio.2016.05.027 27212425

[B80] Nordsletten D. Capilnasiu A. Zhang W. Wittgenstein A. Hadjicharalambous M. Sommer G. (2021). A viscoelastic model for human myocardium. Acta Biomater. 135, 441–457. 10.1016/j.actbio.2021.08.036 34487858

[B81] O'Brien F. J. Harley B. A. Yannas I. V. Gibson L. J. (2005). The effect of pore size on cell adhesion in collagen-GAG scaffolds. Biomaterials 26 (4), 433–441. 10.1016/j.biomaterials.2004.02.052 15275817

[B82] O’Leary C. Cavanagh B. Unger R. E. Kirkpatrick C. J. O’Dea S. O’Brien F. J. (2016). The development of a tissue-engineered tracheobronchial epithelial model using a bilayered collagen-hyaluronate scaffold. Biomaterials 85, 111–127. 10.1016/j.biomaterials.2016.01.065 26871888

[B83] Palaveniene A. Tamburaci S. Kimna C. Glambaite K. Baniukaitiene O. Tihminlioğlu F. (2019). Osteoconductive 3D porous composite scaffold from regenerated cellulose and cuttlebone-derived hydroxyapatite. J. biomaterials Appl. 33 (6), 876–890. 10.1177/0885328218811040 30451067

[B84] Park H. S. Lee J. S. Jung H. Kim D. Y. Kim S. W. Sultan M. T. (2018a). An omentum-cultured 3D-printed artificial trachea: *in vivo* bioreactor. Artif. Cells Nanomedicine Biotechnol. 46 (Suppl. 3), 1131–1140. 10.1080/21691401.2018.1533844 30451550

[B85] Park H. S. Park H. J. Lee J. Kim P. Lee J. S. Lee Y. J. (2018b). A 4-Axis technique for Three-Dimensional printing of an artificial trachea. Tissue Eng. Regen. Med. 15 (4), 415–425. 10.1007/s13770-018-0136-8 30603565 PMC6171658

[B86] Perez R. A. Mestres G. (2016). Role of pore size and morphology in musculo-skeletal tissue regeneration. Mater. Sci. Eng. C 61, 922–939. 10.1016/j.msec.2015.12.087 26838923

[B87] Pezhouman A. Nguyen N. B. Kay M. Kanjilal B. Noshadi I. Ardehali R. (2023). Cardiac regeneration–Past advancements, current challenges, and future directions. J. Mol. Cell. Cardiol. 182, 75–85. 10.1016/j.yjmcc.2023.07.009 37482238

[B88] Pham Q. P. Sharma U. Mikos A. G. (2006). Electrospun poly(ε-caprolactone) microfiber and multilayer nanofiber/microfiber Scaffolds: characterization of scaffolds and measurement of cellular infiltration. Biomacromolecules 7 (10), 2796–2805. 10.1021/bm060680j 17025355

[B89] Pok S. Myers J. D. Madihally S. V. Jacot J. G. (2013). A multilayered scaffold of a chitosan and gelatin hydrogel supported by a PCL core for cardiac tissue engineering. Acta Biomater. 9 (3), 5630–5642. 10.1016/j.actbio.2012.10.032 23128158 PMC3562398

[B90] Ramasamy S. Davoodi P. Vijayavenkataraman S. Teoh J. H. Thamizhchelvan A. M. Robinson K. S. (2021). Optimized construction of a full thickness human skin equivalent using 3D bioprinting and a PCL/collagen dermal scaffold. Bioprinting 21, e00123. 10.1016/j.bprint.2020.e00123

[B91] Rouwkema J. Rivron N. C. van Blitterswijk C. A. (2008). Vascularization in tissue engineering. Trends Biotechnol. 26 (8), 434–441. 10.1016/j.tibtech.2008.04.009 18585808

[B92] Sabree I. Gough J. E. Derby B. (2015). Mechanical properties of porous ceramic scaffolds: influence of internal dimensions. Ceram. Int. 41 (7), 8425–8432. 10.1016/j.ceramint.2015.03.044

[B93] Sadeghi A. Zandi M. Pezeshki-Modaress M. Rajabi S. (2019). Tough, hybrid chondroitin sulfate nanofibers as a promising scaffold for skin tissue engineering. Int. J. Biol. Macromol. 132, 63–75. 10.1016/j.ijbiomac.2019.03.208 30928369

[B94] Shahin-Shamsabadi A. Hashemi A. Tahriri M. Bastami F. Salehi M. Mashhadi Abbas F. (2018). Mechanical, material, and biological study of a PCL/bioactive glass bone scaffold: importance of viscoelasticity. Mater. Sci. Eng. C 90, 280–288. 10.1016/j.msec.2018.04.080 29853093

[B95] Shakir S. Hackett T. L. Mostaço-Guidolin L. B. (2022). Bioengineering lungs: an overview of current methods, requirements, and challenges for constructing scaffolds. Front. Bioeng. Biotechnol. 10, 1011800. 10.3389/fbioe.2022.1011800 36394026 PMC9649450

[B96] Shou Y. Teo X. Y. Wu K. Z. Bai B. Kumar A. R. K. Low J. (2023). Dynamic stimulations with bioengineered extracellular matrix-mimicking hydrogels for mechano cell reprogramming and therapy. Adv. Sci. Weinheim, Baden-Wurttemberg, Ger. 10 (21), e2300670. 10.1002/advs.202300670 PMC10375194 37119518

[B97] Smit T. Huyghe J. M. Cowin S. C. (2002). Estimation of the poroelastic parameters of cortical bone. J. Biomechanics 35 (6), 829–835. 10.1016/S0021-9290(02)00021-0 12021003

[B98] Söhling N. Neijhoft J. Nienhaus V. Acker V. Harbig J. Menz F. (2020). 3D-Printing of hierarchically designed and osteoconductive bone tissue engineering scaffolds. Mater. (Basel) 13 (8), 1836. 10.3390/ma13081836 PMC7215341 32295064

[B99] Sumathy B. Nair P. D. (2020). Keratinocytes-hair follicle bulge stem cells-fibroblasts co-cultures on a tri-layer skin equivalent derived from gelatin/PEG methacrylate nanofibers. J. biomaterials Sci. 31 (7), 869–894. 10.1080/09205063.2020.1725861 32028856

[B100] Sumathy B. Velayudhan S. (2023). Fabrication and evaluation of a bi-layered gelatin based scaffold with arrayed micro-pits for full-thickness skin construct. Int. J. Biol. Macromol. 251, 126360. 10.1016/j.ijbiomac.2023.126360 37591428

[B101] Sun Y. Hsu C. C. Ling T. Liu L. Lin T. Jakfar S. (2020). The preparation of cell-containing microbubble scaffolds to mimic alveoli structure as a 3D drug-screening system for lung cancer. Biofabrication 12 (2), 025031. 10.1088/1758-5090/ab78ee 32084662

[B102] Tadevosyan K. Iglesias-García O. Mazo M. M. Prósper F. Raya A. (2021). Engineering and assessing cardiac tissue complexity. Int. J. Mol. Sci. 22 (3), 1479. 10.3390/ijms22031479 33540699 PMC7867236

[B103] Tamimi M. Rajabi S. Pezeshki-Modaress M. (2020). Cardiac ECM/chitosan/alginate ternary scaffolds for cardiac tissue engineering application. Int. J. Biol. Macromol. 164, 389–402. 10.1016/j.ijbiomac.2020.07.134 32702419

[B104] Tavakoli M. Mirhaj M. Varshosaz J. Salehi S. Mohanna S. M. Salehi S. (2023). Asymmetric tri-layer sponge-nanofiber wound dressing containing insulin-like growth factor-1 and multi-walled carbon nanotubes for acceleration of full-thickness wound healing. Biomater. Adv. 151, 213468. 10.1016/j.bioadv.2023.213468 37220673

[B105] Tenreiro M. F. Louro A. F. Alves P. M. Serra M. (2021). Next generation of heart regenerative therapies: progress and promise of cardiac tissue engineering. npj Regen. Med. 6, 30. 10.1038/s41536-021-00140-4 34075050 PMC8169890

[B106] Thomson K. S. Korte F. S. Giachelli C. M. Ratner B. D. Regnier M. Scatena M. (2013). Prevascularized microtemplated fibrin scaffolds for cardiac tissue engineering applications. Part A 19 (7-8), 967–977. 10.1089/ten.tea.2012.0286 PMC3589898 23317311

[B107] Townsley M. I. (2012). Structure and composition of pulmonary arteries, capillaries, and veins. Compr. Physiol. 2, 675–709. 10.1002/cphy.c100081 23606929 PMC3630377

[B108] Trifonov A. Shehzad A. Mukasheva F. Moazzam M. Akilbekova D. (2024). Reasoning on pore terminology in 3D bioprinting. Gels 10 (2), 153. 10.3390/gels10020153 38391483 PMC10887720

[B109] Tytgat L. Kollert M. R. Damme L. V. Thienpont H. Ottevaere H. Duda G. N. (2020). Evaluation of 3D printed gelatin-based scaffolds with varying pore size for MSC-based adipose tissue engineering. Macromol. Biosci. 20 (4), 1900364. 10.1002/mabi.201900364 32077631

[B110] Vagnozzi R. J. Maillet M. Sargent M. A. Khalil H. Johansen A. K. Z. Schwanekamp J. A. (2020). An acute immune response underlies the benefit of cardiac stem cell therapy. Nature 577 (7790), 405–409. 10.1038/s41586-019-1802-2 31775156 PMC6962570

[B111] Vunjak-Novakovic G. Tandon N. Godier A. Maidhof R. Marsano A. Martens T. P. (2010). Challenges in cardiac tissue engineering. Tissue Eng. Part B Rev. 16 (2), 169–187. 10.1089/ten.teb.2009.0352 19698068 PMC2946883

[B112] Wang L. Zhao Y. Yang F. Feng M. Zhao Y. Chen X. (2020). Biomimetic collagen biomaterial induces *in situ* lung regeneration by forming functional alveolar. Biomaterials 236, 119825. 10.1016/j.biomaterials.2020.119825 32044576

[B113] Wang W. Tao H. Zhao Y. Sun X. Tang J. Selomulya C. (2017). Implantable and biodegradable macroporous iron oxide frameworks for efficient regeneration and repair of infracted heart. Theranostics 7 (7), 1966–1975. 10.7150/thno.16866 28638482 PMC5479283

[B114] Weibel E. R. (1970). Morphometric estimation of pulmonary diffusion capacity. Respir. Physiol. 11 (1), 54–75. 10.1016/0034-5687(70)90102-7 4992513

[B115] Weibel E. R. (2009). What makes a good lung? Schweiz. Med. Wochenschr. 139 (27-28), 375–386. 10.4414/smw.2009.12270 19629765

[B116] Xia P. Luo Y. (2022). Vascularization in tissue engineering: the architecture cues of pores in scaffolds. J. Biomed. Mater. Res. Part B Appl. Biomaterials 110 (5), 1206–1214. 10.1002/jbm.b.34979 34860454

[B117] Xu Y. Dai J. Zhu X. Cao R. Song N. Liu M. (2021). Biomimetic trachea engineering via a modular ring strategy based on Bone-Marrow stem cells and atelocollagen for use in extensive tracheal reconstruction. Adv. Mater. 34 (6), e2106755. 10.1002/adma.202106755 34741771

[B118] Xue N. Ding X. Huang R. Jiang R. Huang H. Pan X. (2022). Bone tissue engineering in the treatment of bone defects. Pharm. (Basel) 15 (7), 879. 10.3390/ph15070879 PMC9324138 35890177

[B119] Yadav P. Beniwal G. Saxena K. K. (2020). A review on pore and porosity in tissue engineering. Mater. Today Proc. 44, 2623–2628. 10.1016/j.matpr.2020.12.661

[B120] Yang W. Xu H. Lan Y. Zhu Q. Liu Y. Huang S. (2019a). Preparation and characterisation of a novel silk fibroin/hyaluronic acid/sodium alginate scaffold for skin repair. Int. J. Biol. Macromol. 130, 58–67. 10.1016/j.ijbiomac.2019.02.120 30797808

[B121] Yang Y. Lei D. Zou H. Huang S. Yang Q. Li S. (2019b). Hybrid electrospun rapamycin-loaded small-diameter decellularized vascular grafts effectively inhibit intimal hyperplasia. Acta biomater. 97, 321–332. 10.1016/j.actbio.2019.06.037 31523025

[B122] Yannas I. V. Lee E. Orgill D. P. Skrabut E. M. Murphy G. F. (1989). Synthesis and characterization of a model extracellular matrix that induces partial regeneration of adult mammalian skin. Proc. Natl. Acad. Sci. U. S. A. 86 (3), 933–937. 10.1073/pnas.86.3.933 2915988 PMC286593

[B123] Zandi N. Dolatyar B. Lotfi R. Shallageh Y. Shokrgozar M. A. Tamjid E. (2021). Biomimetic nanoengineered scaffold for enhanced full-thickness cutaneous wound healing. Acta biomater. 124, 191–204. 10.1016/j.actbio.2021.01.029 33508511

[B124] Zhang Y. Sun N. Zhu M. Qiu Q. Zhao P. Zheng C. (2022a). The contribution of pore size and porosity of 3D printed porous titanium scaffolds to osteogenesis. Biomater. Adv. 133, 112651. 10.1016/j.msec.2022.112651 35034817

[B125] Zhang Y. Zhang M. Cheng D. Xu S. Du C. Xie L. (2022b). Applications of electrospun scaffolds with enlarged pores in tissue engineering. Biomaterials Sci. 10 (6), 1423–1447. 10.1039/D1BM01651B 35170597

[B126] Zhao F. Xiong Y. Ito K. van Rietbergen B. Hofmann S. (2021). Porous geometry guided micro-mechanical environment within scaffolds for cell mechanobiology study in bone tissue engineering. Front. Bioeng. Biotechnol. 9, 736489. 10.3389/fbioe.2021.736489 34595161 PMC8476750

[B127] Zhao Y. Tan K. Zhou Y. Ye Z. Tan W.-S. (2016). A combinatorial variation in surface chemistry and pore size of three-dimensional porous poly(ε-caprolactone) scaffolds modulates the behaviors of mesenchymal stem cells. Mater. Sci. Eng. C 59, 193–202. 10.1016/j.msec.2015.10.017 26652364

[B128] Zhong L. Chen J. Ma Z. Feng H. Chen S. Cai H. (2020). 3D printing of metal–organic framework incorporated porous scaffolds to promote osteogenic differentiation and bone regeneration. Nanoscale 12 (48), 24437–24449. 10.1039/D0NR06297A 33305769

[B129] Zimmermann W. H. Didié M. Döker S. Melnychenko I. Naito H. Rogge C. (2006). Heart muscle engineering: an update on cardiac muscle replacement therapy. Cardiovasc. Res. 71 (3), 419–429. 10.1016/j.cardiores.2006.03.023 16697358

[B130] Zou F. Zhao N. Fu X. Diao J. Ma Y. Cao X. (2016). Enhanced osteogenic differentiation and biomineralization in mouse mesenchymal stromal cells on a β-TCP robocast scaffold modified with collagen nanofibers. RSC Adv. 6 (28), 23588–23598. 10.1039/C5RA26670J

